# HSP47 is a potential dual cell target and prognostic factor in pancreatic cancer

**DOI:** 10.1038/s41388-026-03865-y

**Published:** 2026-06-21

**Authors:** George Sharbeen, Estrella Gonzales-Aloy, Janet Youkhana, John Kokkinos, Rosa Mistica C. Ignacio, Joshua A. McCarroll, Aparna S. Raina, Chantal Kopecky, Jie Liu, Amanda Mawson, Andrea Nunez, Cyrille Boyer, Mert Erkan, David Goldstein, Koroush S. Haghighi, Paul Timpson, Sean C. Warren, Kendelle J. Murphy, Anthony J. Gill, Amber L. Johns, Angela Chou, Elvis Pandzic, Sandra Fok, Renee Whan, Val Gebski, Jennifer P. Morton, Minoti Apte, Phoebe A. Phillips

**Affiliations:** 1 Pancreatic Cancer Translational Research Group, School of Biomedical Sciences, Lowy Cancer Research Centre, UNSW Sydney, Sydney, NSW Australia; 2 Australian Centre for Nanomedicine, UNSW Sydney, Sydney, NSW Australia; 3 Children’s Cancer Institute, Health Translation Hub, UNSW Sydney, Sydney, NSW Australia; 4 UNSW RNA Institute, UNSW Sydney, Sydney, NSW Australia; 5 The Garvan Institute of Medical Research and the Kinghorn Cancer Centre, Sydney, NSW Australia; 6 School of Chemical Engineering, UNSW Sydney, Sydney, NSW Australia; 7 Mehmet Ali Aydinlar Acibadem University, Department of General Surgery; Ataşehir Hospital, Istanbul, Turkey; 8 School of Clinical Medicine, UNSW Sydney, Prince of Wales Hospital, Sydney, NSW Australia; 9 Tumour Ecosystems Program, The Garvan Institute of Medical Research and the Kinghorn Cancer Centre, Sydney, NSW Australia; 10 Australian Pancreatic Cancer Genome Initiative (APGI), Sydney, NSW Australia; 11 St Vincents School of Clinical Medicine, Faculty of Medicine and Health, UNSW Sydney, Sydney, NSW Australia; 12 Cancer Diagnosis and Pathology Group, Kolling Institute of Medical Research, Royal North Shore Hospital, University of Sydney, Sydney, NSW Australia; 13 Department of Anatomical Pathology, Royal North Shore Hospital, University of Sydney, Sydney, NSW Australia; 14 Katharina Gaus Light Microscopy Facility, Mark Wainwright Analytical Centre, Lowy Cancer Research Centre, UNSW Sydney, Sydney, NSW Australia; 15 NHMRC Clinical Trials Centre, University of Sydney, Sydney, NSW Australia; 16 School of Cancer Sciences, University of Glasgow, Glasgow UK and CRUK Scotland Institute, Glasgow, UK; 17 Pancreatic Research Group, South Western Sydney Clinical Campuses, School of Clinical Medicine, UNSW and Ingham Institute for Applied Medical Research, Sydney, NSW, Australia

**Keywords:** Pancreatic cancer, Cancer microenvironment

## Abstract

Pancreatic ductal adenocarcinoma (PDAC) is a deadly disease with a dismal 5-year survival rate at 12%. The fibrotic PDAC desmoplasia is a major contributor to chemoresistance and metastasis that drive this poor prognosis. Cancer-associated fibroblasts (CAFs) generate PDAC tumour fibrosis and have been identified as therapeutic targets to remodel the stroma to a more drug-permissive microenvironment. We assessed the therapeutic potential of inhibiting the collagen chaperone, heat shock protein 47 (HSP47) in PDAC cells and CAFs. Collagen is a key component of PDAC fibrosis and requires the activity of HSP47 to ensure correct maturation and secretion. Herein, we show that HSP47 knockdown inhibits both PDAC cells and CAF proliferation in vitro. In vivo, therapeutic HSP47 knockdown in orthotopic PDAC tumours significantly reduced intratumoural fibrosis and opened intratumoural blood vessels, while stable HSP47 knockdown specifically in CAFs additionally reduced PDAC tumour growth. We observed that HSP47 is highly expressed in the stroma of >80% of patients in a PDAC cohort (Australian Pancreatic Cancer Genome Initiative), but that it was only prognostic of poorer overall survival in the tumour compartment. Functional relevance in the tumour compartment was further validated in 3D human PDAC explants. Our work demonstrates that HSP47 is a potential therapeutic target in both PDAC cells and CAFs and represents a robust target to interfere with tumour collagen deposition.

## Introduction

Pancreatic ductal adenocarcinoma (PDAC) is a lethal malignancy that is predicted to become the 2nd leading cause of cancer-related death by 2030 [1, 2]. The majority of PDAC patients present late and rely on systemic chemotherapy, but these therapeutics only extend median survival by a matter of months. A major contributor to PDAC chemoresistance is the characteristic stromal fibrosis, which can constitute up to 90% of the tumour volume [3–5]. Fibrosis distorts tumour vasculature, creating a physical barrier to drug delivery [3–5] but also creates oxygen-deprived and nutrient-deprived conditions that drive the epithelial-to-mesenchymal transition of PDAC cells, which is a more chemoresistant phenotype [3–5]. Components of PDAC fibrosis have also been shown to be directly involved in creating a microenvironment that promotes PDAC cell invasion and metastasis [6]. Murphy et al. [7] demonstrated that resectable tumours from PDAC patients that received pre-surgery gemcitabine and Abraxane neoadjuvant, had significantly higher fibrosis, indicating fibrosis is also an early tumour survival response to chemotherapeutic treatment. This makes it important to remodel fibrosis to create a more drug-permissive tumour microenvironment. PDAC fibrosis is generated by cancer-associated fibroblasts (CAFs; [3–5]). CAFs are resident fibroblasts that are hijacked from PDAC cells, resulting in the secretion of excessive extracellular matrix proteins and pro-tumour signals [3–5]. Several studies have now identified specific CAF subsets that express markers associated with distinct functions [8, 9]. The CAF subset that has been attributed with stromal remodelling roles are myofibroblasts CAFs (myCAFs; herein referred to as CAFs) highly expressing α-smooth muscle actin (αSMA) [8, 9]. Due to the complexity of the CAF compartment, stromal ablation can be detrimental to therapeutic efficacy [10–13]. This highlights the need to specifically target tumour-promoting components of the PDAC stroma.

Heat shock protein 47 (HSP47, also known as serpinh1, gp46) represents a potential avenue to remodel PDAC stroma to a more drug-permissive state. HSP47 is a collagen chaperone that plays a critical role in correcting folding, maturation and secretion of collagen, a key component of fibrosis [14]. It has consequently drawn interest as a potential therapeutic target in a variety of fibrotic conditions and fibrotic cancers [15]. In one of the earliest studies of HSP47 expression in human PDAC, Maitra et al. [16] demonstrated that HSP47 was highly expressed in the stromal desmoplasia of 57 PDAC patients and interestingly, in the epithelial compartment of 65% of these patients, but was absent from normal pancreatic tissue. Iacobuzio-Donahue et al. [17] similarly showed 6-fold upregulation of HSP47 transcripts in PDAC versus normal pancreas. These findings have since been confirmed by studies at both the mRNA level [18] and in PDAC tissue microarrays [19]. Ishiwatari et al. [20] demonstrated that knocking down HSP47 in a model of chemically induced pancreatic fibrosis using vitamin-A-coupled nanoparticle delivery of HSP47-siRNA could alleviate fibrosis. Han et al. [21] subsequently showed this approach could reduce intratumoural fibrosis and sensitise PDAC tumours to gemcitabine [21].

Our study aimed to understand the relevance of HSP47 as a dual cell therapeutic target and to better understand functional relevance in PDAC cells versus CAFs. Interestingly, HSP47 has also been shown to play a direct pro-survival role in PDAC cells [22, 23]. Chen et al. [24] highlighted the pro-tumour role of homotrimeric collagen specifically produced by PDAC cells and not CAFs. Herein, we show that knockdown of HSP47 in PDAC cells and CAFs has therapeutic potential in both cell types. HSP47 knockdown in PDAC cells and CAFs reduced their proliferation in vitro, reduced PDAC cell frequency in 3D pancreatic tumour explants, and hindered organoid formation when knocked down in CAFs. We also demonstrate that we can therapeutically reduce intratumoural fibrosis in orthotopic PDAC tumours in mice using an organic nanoparticle (Star 3; [25]) to systemically deliver HSP47-siRNA to PDAC tumours. Finally, we demonstrate that high HSP47 expression in the PDAC tumour compartment, but not the stromal compartment, is prognostic of poorer overall survival and is associated with higher expression of poorly organised collagen.

## Methods

### Sex as a biological variable

Pancreatic cancer affects both sexes in almost equal proportions. Our clinical cohort analysis included both male and female patients. In vivo studies used female athymic host mice. Sex in this in vivo model does not affect therapeutic response.

### Cell culture

Human pancreatic cancer cell lines (MiaPaca-2, Panc-1, AsPC1) were purchased from ATCC and were cultured as previously described [25–27]. Human CAFs were isolated from PDAC tumour tissue by explant culture (using outgrowth method) and were utilised up to 12 passages [28, 29]. CAF purity was assessed by 100% positive immunostaining for alpha-smooth muscle actin (αSMA) and glial fibrillary acidic protein (GFAP) and negative immunostaining for cytokeratin as described [30]. IMDM media with 10% FBS, 4mM L-glutamine was used to culture all CAFs. All cells were continued in culture using humidified incubator (37 °C, 5% CO_2_) and tested negative for mycoplasma [MycoAlert Mycoplasma Detection kit (Lonza)] once monthly throughout study. The use of human derived CAFs were approved by UNSW Sydney human ethics committee (approvals: HC14039, HC180973, HREC13/023) and the German Technical University of Munich human ethics committee, approval:5510/12).

### Western blot for HSP47

Protein lysates were prepared, quantified, and electrophoresed on a 10% SDS-PAGE gel and nitrocellulose membrane was used for transfer as described [26]. The following antibodies were used to probe the blots: HSP47 (Stressgen, Cat. SPA-470; 1:1000), GAPDH (Abcam, Cat. ab8245; 1:50,000), anti-mouse IgG-HRP secondary antibody (DAKO, Cat. P0447; 1:2000). Bands were visualised using Amersham ECL (RPN2106, GE Healthcare) and scanned using LAS4000 and quantified using ImageJ.

### siRNA transfection

100 nM smartpool On-target plus human HSP47-siRNA pool (Dharmacon Cat. L-011230-00-0020; 4x siRNA sequences) or non-silencing siRNA (ns-siRNA Dharmacon Cat. D-001810-10-20) was utilised to transfect human CAFs, MiaPaCa2, AsPC1 as previously described [25–27].

### Cell cycle microarray

48 h post-transfection with ns-siRNA pool or HSP47-siRNA, CAFs were treated with 10 ng/mL PDGF for 48 h after which RNA was extracted and processed for qPCR array. TaqMan Gene Signature plates for cell cycle were used (AB Applied Biosystem, Cat 4414116 and Cat. 4414123) according to manufacturer instructions. ABI 7900 HT instrument was used to run samples.

### The production and establishment of stable human CAF cells expressing HSP47 shRNA

Lentiviral delivery of a human telomerase construct system (GenTarget, Cat. LVP1131-RP) was used to immortalise human patient-derived CAFs (passage 9). Immortalised cells were selected in puromycin and red fluorescent protein-positive cells sorted on a BD FACs Aria II as previously described [31]. The proliferation of the hTERT-CAFs was comparable to that of the parent cells (Supplementary Fig. 1A). hTERT-immortalised CAFs were then transduced with lentiviral scramble(control)-shRNA and HSP47-shRNA sequence (Origene, Cat. TL316478V). Transduced cells were selected with puromycin and GFP-positive cells sorted on a BD FACS Melody (Supplementary Fig. 1B). Western blot confirmed HSP47 knockdown relative to scramble-shRNA controls. All CAF shRNA cell lines were re-validated by positive immunostaining for αSMA and GFAP and negative immunostaining for cytokeratin and were used within 50 passages of immortalisation.

### Immunofluorescence staining

#### HSP47 localisation

CAFs were seeded onto glass chamber slides and allowed to adhere for 48 h. The slides were then fixed with 4% paraformaldehyde, and permeabilised with 0.5% Triton x-100 for 10 min at room temperature. Slides were blocked in 1% BSA/PBS for 30 min at room. Primary antibody to HSP47 (Stressgen, Cat SPA-470; 1:50) was used overnight at 4 °C, followed by three 5-min washes in 0.1% Tween-20/PBS at room temperature. Slides were then incubated with goat anti-mouse AlexaFlour 488 (Invitrogen, Cat. A11001; 1:1000 dilution for 45 min at room temperature), for nuclear stain propidium iodide (Sigma, Cat. P4864; 1:1000 for 5-min at room temperature) was used. Images were captured using Leica confocal microscope.

#### Collagen I staining

CAFs were seeded onto glass chamber slides, 24 h post-seeding, cells were treated with 5mM L-ascorbic acid for 48 h. Cells were then fixed and stained as per HSP47 immunofluorescence procedure above except cells were not permeabilised to maintain extracellular collagen and they were blocked with 2% Goat serum/10% glycerol in PBS for 30 min at room temperature. The follow antibodies were used: Rabbit Collagen type I antibody (Rockland antibodies and assays Cat. 600-401-103-0.5; 1:100) overnight at 4 °C, goat anti-rabbit AlexaFluor 488 (Molecular Probes, Cat. A-11008; 1:1000) 45 min at room temperature. Cells were mounted using Prolong Gold Antifade (ThermoFisher Scientific, Cat. P36931) and imaged using Zeiss 900 confocal microscope.

### Cell proliferation assay

CAF proliferation or viability was measured by cell counting kit-8 (CCK8) kit (Dojindo) or trypan blue as previously described [25–27]. Proliferation and viability measurements were performed at the following time points: i) 72 h post-transfection with siRNA; ii) 48 h post platelet-derived growth factor (PDGF; BioScientific Pty Ltd, Cat. 220-BB; 10 ng/mL), Transforming growth factor beta 1 (TGFβ1; BioScientific Pty Ltd, Cat. HZ-101; 1 ng/mL), or tert-butyl hydroperoxide (tBHP; sigma; 0–40 µM) treatment. MiaPaCa-2 and AsPC1 proliferation were measured by direct cell count (cell-by-cell detection over 16 fields of view per well; average count per field of view graphed) on an IncuCyteS3 platform.

### Cell migration assay

CAF migration was studied using a Boyden Chamber assay as previously described [29] with some modifications. Briefly, 48 h post-transfection with ns-siRNA pool or HSP47-siRNA pool cells were re-seeded into inserts (In-vitro Technologies, Cat. FAL353182) with porous membrane (8.0-µm) placed into wells containing serum-reduced media (0.1% FBS IMDM) ± PDGF (10 ng/mL). Cells were cultured for a further 48 h under these conditions. The number of cell migrated through the pores was expressed as a migration index (%): (number of cells on the bottom of the membrane/total number of cells on both surfaces of the membrane) × 100.

### Cell cycle and apoptosis assays

#### Detection of apoptosis

72 h post siRNA transfection, attached and floating cells were collected, and detection of total apoptosis was performed using an Annexin V-PE/7AAD kit (BD Bioscience, Cat 559763) according to manufacturer’s instruction. BD LSR Fortessa^TM^ was used to analyse data (gating strategy in Supplementary Fig. 1C).

#### Cell cycle assay

48 h post siRNA transfection, CAF cells were treated ±PDGF (10 ng/mL) for 48 h, and cell cycle measured by PI staining and flow cytometry on a BD LSR Fortesa^TM^, as described [26, 27] (gating strategy in Supplementary Fig. 1D).

### Star nanoparticle synthesis

Star nanoparticles (Star 3) were synthesised as previously described by our team [25]. The star nanoparticle was purified by several precipitation in mixture diethyl ether/ hexane to remove unreacted arms. The purified star nanoparticle was analysed by GPC, NMR and FTIR after purification to determine its composition [final composition: foligoethylene glycol methyl ether methacrylate (OEGMA)/fDimethylaminoethyl methacrylate (DMEAMA) 14.5/85.5 mol %; Mn = 155,000 g/mol ( ± 5000 g/mol)]. The nanoparticle was solubilised in methanol and dialysed with acidic water (pH = 3.0) for 24 h, and then further dialysed using water (pH = 6.5) for 48 h, then freeze-dried [Teo et al., Biomacromolecules, 2016]. Star nanoparticle was analysed by DLS [Average Size DLS = 28 (+/−5 nm), Average Zeta potential = 40 (+/−3)]. siRNA encapsulation efficiency at the ratio used (16:1 siRNA:Star 3) is ~100% by gel shift assay.

### Orthotopic pancreatic cancer mouse models

Female Balb/c nude mice (8 weeks old) were used at surgeries. Star 3-siRNA therapeutic study (therapeutic knockdown): 1 × 10^6^ Luciferase-expressing MiaPaCa2 cells and 1 × 10^6^ CAF cells were co-implanted into the tail of the pancreas of mice as previously described [26, 31]. Mice were randomised 4 weeks post-surgery based on luminescence as previously described [26, 31]. Blinding was not possible during treatments due to staff availability. Mice were then treated with 3 mg/kg control-siRNA (antisense: 5’-GAACUUCAGGGUCAGCUUGCCG) or HSP47ss-siRNA single sequence (antisense: 5’-AGCCCAGCCAGGUGUUUCUUU) complexed to Star 3 intravenously daily for the first three days, followed by twice weekly for 4 weeks. Orthotopic model (stable shRNA cell lines): 1 × 10^6^ Luciferase-expressing MiaPaCa2 cells and 1 × 10^6^ immortalised CAFs stably expressing either control-shRNA or HSP47-shRNA, were co-implanted into the tail of the pancreas as previously described [26, 31]. VisualSonics Vevo 3100 ultrasound was used to measure tumour volume weekly from week 4 post-implantation. Mice with no measurable tumour at week 4 were excluded (2 exclusions). At the end of both studies, mice were humanely euthanised and organs/tumours harvested. Calliper measurement was carried out to calculate tumour volume (Length × Width × Height/2), with operator blinded to treatment. Tumours were fixed in 4% paraformaldehyde and paraffin embedded. The detection of metastases was conducted by ex vivo luminescence (>600 counts) and confirmed by H&E as previously described [26, 31].

### Polarised light analysis in tumour sections

Polarised light imaging of picrosirius red-stained tissue sections was performed on an Olympus VS200 slide scanner using a 40×/0.95 objective. The percentages of low-, medium-, and high-birefringent collagen were calculated from scanned whole explant sections using the MATLAB Colour Thresholder application. The analysis was performed on HSV-converted images, where Hue (H), Saturation (S), and Value (V) range from 0 to 1. Hue represents a circular colour wheel starting at 0 (red) and progressing through yellow, green, cyan, blue, magenta, and back to red at 1. For classification, Hue-Saturation-Value thresholding was applied as follows: High birefringent (red) fibers: 0.9 ≤ H ≤ 1.066, equivalent to ~324°–23.7° on the 0–360° scale, covering raspberry to orange hues including reds. Medium birefringent (orange) fibers: 0.0661 ≤ H ≤ 1.149, corresponding to 23.7°–53.6°, which spans orange to yellow hues. Low birefringent (green) fibers: 1.1491 ≤ H ≤ 1.398, equivalent to 53.6°–143°, covering yellow-green to turquoise hues. These ranges encompass the major hues observed in Picrosirius Red polarization images. Saturation was not restricted (0–1) to avoid bias from fiber brightness variations, which can occur due to sample thickness rather than actual birefringence. A Value threshold of V > 0.113 was applied to exclude background pixels outside tissue regions.

### Immunohistochemistry and histology for Alpha Smooth Muscle Actin (αSMA), Picrosirius red, and CD31 in mouse PDAC tumours

Paraformaldehyde-fixed, paraffin-embedded tumour sections (5 µm) were stained by immunohistochemistry as described [26, 27, 31]. For mouse primary antibodies Mouse on Mouse (M.O.M) immunodetection kit (Vector Laboratories, Cat. BMK-2202) was used to stain mouse PDAC tumours following manufacturer’s instructions. The following primary antibodies were used: mouse αSMA (1:5000) (Sigma Cat. A5228), and rat CD31 (1:20) (Dianova, Cat. DIA-310). Secondary antibody for CD31 was Biotinylated Rabbit anti-rat IgG (Vector Laboratories, Cat. BA-4000; 1:100). Hematoxylin was used as a counter stain and 3, 3′ diaminobenzidine tetrahydrochloride (DAB) substrate was used to visualise immunohistochemistry staining as previously described [26, 27, 31]. CD31-positive blood vessels (manual count of open vs closed) and αSMA area coverage were analysed in representative tumour regions using ImageJ with colour deconvolution module or in Qupath using positive pixel detection (excluding necrotic regions). The average tumour coverage of representative regions was 24% for αSMA analysis and 53% for CD31 analysis. For Picrosirius red histology, 5 µm paraffin-embedded tumour sections were stained with 0.1% picrosirius red and counter-stained with methyl green or hematoxylin. Representative region average tumour coverage was 55%. All tissue sections were scanned on AperioXT (Lecia Biosystems) or Vectra Polaris (PerkinElmer) slide scanner. QuPath software and ImageJ were used to quantify the amount of αSMA and CD31 positivity in the whole-tissue.

### 3D organoid growth assay

Co-culture organoids were established by mixing MiaPaCa-2 PDAC cells and human CAFs in a 1:1 ratio (5000 cells total), allowing spheroid formation for 24 h in a low adhesion, round bottom 96-well plate as previously described [31], then embedded in 100 μL 1:1 mix of Matrigel (ref) and complete culture medium (IMDM, 4mM L-glutamine, 10% FBS). Growth of spheroids was monitored over 4 days by bright field photos and area measurement, and standardised back to day 0 spheroid area. For organoids involving shRNA-expression CAFs, these cells were co-seeded with parent MiaPaCa-2 cells immediately from general culture. For organoids involving siRNA transfection of CAFs and MiaPaCa-2 cells, cells were transfected with ns-siRNA or HSP47-siRNA as above, then co-seeded to form spheroids 24 h post-transfection.

### Contraction assay

Organotypic matrices were generated as previously described [6, 32]. Here, 3.5 × 10^4^ CAFs/matrix were embedded into acid-extracted rat tail collagen (~2 mg/ml) in the presence of 1× MEM, 8.8% FBS and neutralized with sodium hydroxide. Matrices were allowed to set at 37 °C prior to detachment and then allowed to contract for 6 days. For siRNA treatment, CAFs were transfected with ns-siRNA or HSP47-siRNA as detailed above, 24 h pre-embedding into collagen plugs. For shRNA treatment, CAFs stably expressing scramble or HSP47 shRNA were immediately embedded int collagen plugs.

### HSP47 survival correlation in human PDAC cohort

Formalin-fixed, paraffin-embedded human PDAC tissue microarrays (TMAs) were received from the Australian Pancreatic Cancer Genome Initiative (APGI; International Cancer Genome Consortium Cohort and Training cohort; patient demographics in Table 1) with written informed consent from patients. Tissues were stained with HSP47 by immunohistochemistry as previously described [26, 27, 31]. Briefly, primary HSP47 antibody was used at 1:50 dilution, sections were incubated with primary antibody for 1 h at room temperature while secondary antibody used was biotinylated Goat anti-mouse (DAKO, Cat. E0433) at 1:200 dilution for 45 min at room temperature. Survival analyses were performed as previously described [31]. Staining intensity was scored by two independent scorers in tumour and stromal compartments. Scoring for stain intensity was conducted using a four-point scale (0–3), based on staining intensity in ≥75% of each compartment (normal acinar/ductal cells excluded). A consensus score was obtained for each core. The highest tumour and stroma scores were used for each set of 3 cores per patient. Scores were then dichotomised into HSP47^low^ (scores of 0–1) and HSP47^high^ (scores of 2–3) groups for each compartment. Kaplan–Meier Survival Curve was then used to correlate scores with overall survival. Survival for HSP47^low^ vs HSP47^high^ groups was compared for individual tumour and stromal compartments, and for combined tumour and stromal scores for each patient. Patients were censored if they were deceased due to other causes/still alive, in addition non-PDAC tumours were also excluded. To correlate HSP47 expression with collagen content (total picrosirius red coverage, second harmonics generation and GLCM) we used data generated by Murphy et al. [7] and Vennin et al. [33] for the APGI PDAC cohort and matched it to each patient.

**Table 1:** Australian Pancreatic Cancer Genome Initiative Training and International Cancer Genome Cohort (ICGC) patient cohort characteristics.

Age at diagnosis	Number of patients	Overall stage	Number of patients
>50	197	IA	0
≤50	20	IB	5
Gender		IIA	58
Male	116	IIB	147
Female	101	III	0
Ethnicity		IV	7
Asian	12	TNM staging	
Asian, White/Caucasian	1	T1	7
Black/African	12	T2	19
Pacific Islander	1	T3	191
White/Caucasian	178	N0	61
Not reported	13		
Smoker		N1	89
Ever	114	N1a	17
Never	94	N1b	47
Not reported	9	NX	3
Alcohol consumption		M0	53
Ever	110	M1	8
Never	93	MX	156
Not reported	14	Perineural invasion	
Margin status		Yes	170
R0	138	No	31
R1	68	Not reported	16
Not reported	11	Vascular invasion	
Macroscopic tumour location		Yes	116
Ampulla	2	No	67
Body	13	Not reported	34
Head	159	Recurrence at liver	
Head (Uncinate)	6	Yes	67
Tail	24	No	81
Not reported	13	No recurrence	69

*TNM* Tumour, Node, Metastasis.

### PDAC tumour explant model

PDAC patient-derived tumour explants were established and treated with Star 3+control-siRNA or Star 3+HSP47ss-siRNA every 72 h (days 0, 3, 6, and 9), as previously described [31, 34]. Day 0 and day 12 explants were fixed in 4% paraformaldehyde, paraffin embedded and sectioned as previously described [31, 34]. Histology (H&E, picrosirius red) and immunohistochemistry for HSP47 (Enzo Life Sciences, Cat. SPA-470; 1:100 dilution overnight at 4 °C), cytokeratin (DAKO, Cat. M3515; 1:100 overnight at 4 °C), αSMA (Sigma-Aldrich, Cat. A5228; 1:1000 dilution 1 h room temperature) and bromodeoxyuridine (DAKO, Cat. M0744; 1:50 dilution overnight at 4 °C) (BrdU; cells pulsed for final 24 h of assay with 10 µM BrdU) were performed on explant sections as previously described [31, 34] using the conditions described in immunohistochemistry and survival correlation methods sections above. Frequency of cytokeratin, BrdU and αSMA positive cells was quantified from whole explant sections using QuPATH. Polarised light analysis performed as per orthotopic model analysis.

#### Statistics

Two-tailed Student’s *t* test (2 groups) or one-way/two-way ANOVA (≥3 groups; post-hoc test: Dunn’s, Sidak’s or Bonferroni multiple comparison) were performed for statistical comparison using GraphPad Prism (version 10.4.1). For survival curves, the multivariate associations between variables and time event were created from the Cox proportional hazards (PH) regression and survival curves calculated using the method of Kaplan–Meier (KM). Survival analyses were performed using Analysis of Censored and Correlated Data (ACCoRD) V6.4 Boffin. A *p*-value of ≤0.05 was considered statistically significant. Sample size was selected based on similar prior studies that demonstrated minimum number required to obtain statistical significance for in vitro and in vivo studies. Patient number for the cohort analysis were based on the maximum number of patients provided in the cohort. All specific replicate numbers and individual replicate data points are provided in figures and figure legends. All in vitro experiments utilizing PDAC cells and/or immortalised CAFs were technical replicates. All explants experiments, in vivo experiments and in vitro experiments involving CAFs are biological replicates (Independent patient-derived CAFs).

#### Study approval

In vivo models: All mouse work was approved by the UNSW Sydney animal care and ethics committee (approval: ACEC 16/25B, ACEC 19/3A). Survival correlations: All studies involving the use of human specimens were approved by the UNSW Sydney human ethics committee (approvals: HC14039, HC180973). All patients provided written informed consent through the APGI. Explant model: Human PDAC tumour specimens were obtained from patients undergoing pancreaticoduodenectomy at Prince of Wales Hospital or Prince of Wales Private Hospital, Randwick, NSW, Australia. All patients provided written informed consent through the Health Science Alliance Biobank, all work was approved by UNSW human ethics (HC180973), and all experiments were performed in accordance with the relevant regulations.

## Results

### HSP47 is upregulated in CAFs and is essential in regulating collagen production

**Fig. 1:**
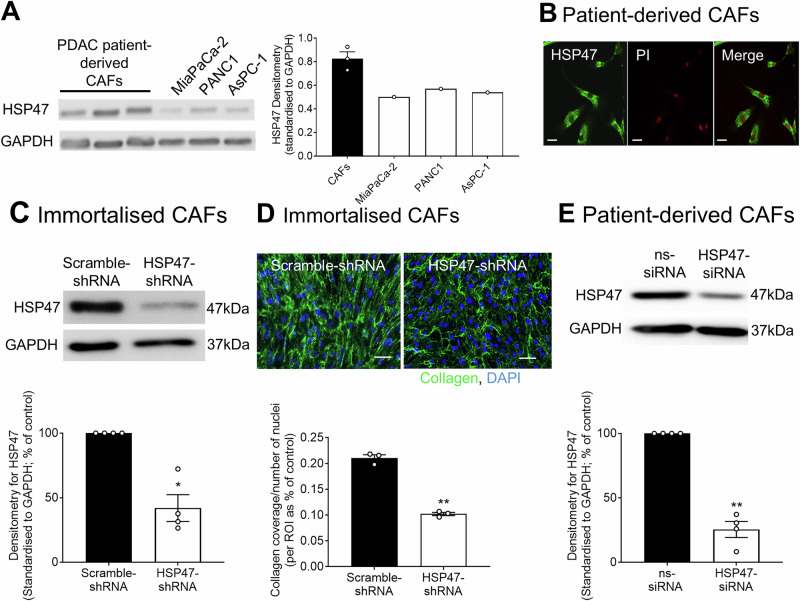
Confirmation of HSP47 upregulation in CAFs and its function regulating collagen production. **A** Western blot comparing HSP47 expression in human PDAC CAFs isolated from 3 independent patients and PDAC cell lines. Graph shows densitometry of protein bands, standardised to GAPDH. **B** Immunofluorescence for HSP47 and PI (nucleus marker) showing HSP47 localisation in the cytoplasm of human CAFs. **C** Western blot of HSP47 in protein extracts from hTERT-immortalised human CAFs stably expressing control(scramble)-shRNA or HSP47-shRNA (*n* = 3). **D** Immunofluorescence for DAPI and collagen in hTERT-immortalised human CAFs stably expressing scramble-shRNA or HSP47-shRNA (*n* = 3). **E** Western blot showing expression of HSP47 protein in human CAFs (*n* = 4). Circles in panels (**A**, **E**) represent biological replicates (independent patient-derived CAFs). Circles in panels (**C**, **D**) indicate technical replicates with the same cell line (**C**, **D**). Bars and lines = mean + s.e.m. Asterisks indicate significance (*p ≤ 0.05, **p ≤ 0.01; Student’s *t* test). Scale bars in all photos = 50 µm.

We first examined HSP47 expression in cultured human patient-derived PDAC CAFs by Western blot and observed significantly higher expression compared to PDAC cell lines, MiaPaCa-2, Panc-1 and AsPC-1 (Fig. 1A). Immunofluorescence staining for HSP47 showed expected localisation in the cytoplasm of CAFs (Fig. 1B). We examined the effect of stable knockdown of HSP47 in CAFs on collagen production. Stable knockdown of HSP47 in hTERT-immortalised CAFs using shRNA was confirmed by Western blot (Fig. 1C). Immunofluorescence staining for collagen αI showed that CAFs expressing HSP47-shRNA had significantly reduced intracellular and extracellular collagen relative to control-shRNA CAFs (46.9 ± 2.1% reduction; Fig. 1D).

### HSP47 knockdown in PDAC cells and CAFs in vitro reduces their proliferation

**Fig. 2:**
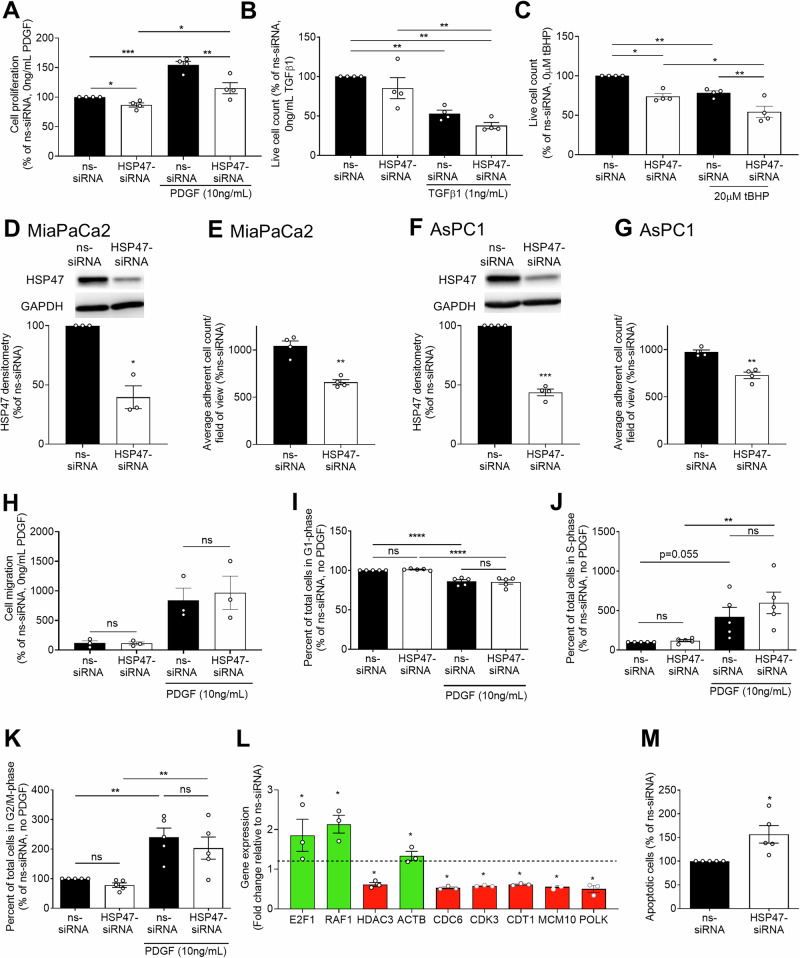
HSP47 knock-down in CAFs in vitro reduces their proliferation and survival in the presence of tumour microenvironment stress conditions and pro-collagen/pro-proliferative cytokines. **A** Cell proliferation (cell counting kit 8) of CAFs 96 h post-transfection with ns-siRNA or HSP47-siRNA ± PDGF (10 ng/mL) (*n* = 4). **B** Live cell counts (trypan blue exclusion assay) of CAFs 96 h post-transfection with ns-siRNA or HSP47-siRNA ± TGFβ1 (1 ng/mL) (*n* = 4). **C** Cell proliferation (trypan blue exclusion assay) of CAFs 72 h post-transfection with ns-siRNA pool or HSP47-siRNA pool ±20–40 µM tert-butyl hydroperoxide (tBHP; oxidative stress) (*n* = 4). **D** Western blot assessment of HSP47 expression in MiaPaCa2 PDAC cancer cell line 72 h post-transfection with ns-siRNA pool or HSP47-siRNA pool (*n* = 3). **E** Adherent cell count (average number of cells per field of view, as measured on an IncuCyte S3 platform) of MiaPaCa2 72 h post-transfection of ns-siRNA or HSP47-siRNA (*n* = 4). **F** Western blot assessment of HSP47 expression in AsPC1 PDAC cells 72 h post-transfection with ns-siRNA or HSP47-siRNA (*n* = 4). **G** Adherent cell count (average number of cells per field of view, as measured on an IncuCyte S3 platform) of AsPC1 cells 72 h post-transfection with ns-siRNA pool or HSP47-siRNA pool (*n* = 4). **H** Quantification of CAF migration 96 h post-transfection with ns-siRNA or HSP47-siRNA ± PDGF (10 ng/mL) treatment (*n* = 3). **I**–**K** Cell cycle analysis of CAFs in G1-phase, S-phase, and G2/M-phase, respectively, 96 h post-transfection of ns-siRNA or HSP47-siRNA ± PDGF (10 ng/mL) treatment (*n* = 5). **L** The top cell cycle-related genes with altered expression in CAFs 96 h post-transfection with HSP47-siRNA + PDGF treatment (10 ng/mL), relative to matched ns-siRNA controls with the same treatments (*n* = 3). Dashed line indicates relative gene expression in ns-siRNA controls. Green bars indicate upregulation and red bars indicate downregulation. **M** The fraction of CAFs that were apoptotic (AnnexinV/DAPI positive) 72 h post-transfection with ns-siRNA or HSP47-siRNA (*n* = 5). Circles in panels (**A**–**C**, **H**–**M**) represent biological replicates (independent patient-derived CAFs). Circles in panels (**D**, **E**, **G**) indicate technical replicates (independent experiments with the same cell line). Lines and bars in all graphs = means + s.e.m. Asterisks in all graphs indicate significance (*p ≤ 0.05, **p ≤ 0.01, ***p ≤ 0.001). ns non-significant. Panels (**A**–**C**, **I**–**K**) Two-way ANOVA; panels (**D**–**G**, **L**–**M**) = Student’s *t* test.

We next assessed whether HSP47 supported the survival of CAFs and PDAC cells. HSP47 knockdown using siRNA in CAFs was confirmed by Western blot (Fig. 1E). Under normal culture conditions, HSP47 knockdown in CAFs significantly decreased their proliferation (Fig. 2A–C) and this was exacerbated by the presence of cytokines produced by PDAC cells and CAFs and by microenvironmental stressors present in the PDAC tumour microenvironment, namely PDGF (stimulates proliferation; Fig. 2A), TGF-β1 (stimulates collagen production; Fig. 2B), and tBHP (oxidative stress; Fig. 2C). Likewise, HSP47 knockdown in MiaPaCa-2 PDAC cells (Fig. 2D, E) and AsPC1 PDAC cells (Fig. 2F, G) significantly reduced cell proliferation.

CAF migration was not significantly affected by HSP47 knockdown (Fig. 2H) nor was cell cycle progression (Fig. 2I–K), though we did observe a trend towards increased percentage of cells in S phase, relative to ns-siRNA controls (Fig. 2J). Cell cycle microarray showed that HSP47 knockdown in CAFs also induced downregulation of pol kappa (a lesion bypass DNA polymerase important for resolving checkpoint inhibition of S phase entry), CDK3 (cyclin-dependent kinase that regulates entry into S-phase) and CDC6 (required for initiation of DNA replication), which may be a consequence of hindered cell cycle progression (Fig. 2L). Finally, HSP47 knockdown alone in CAFs significantly increased apoptosis relative to ns-siRNA controls (56.7% ± 18.4% increase; Fig. 2M).

### Therapeutic HSP47 knockdown in orthotopic PDAC tumours does not affect tumour growth but significantly reduces intratumoural collagen, while increasing intratumoural vasculature

**Fig. 3:**
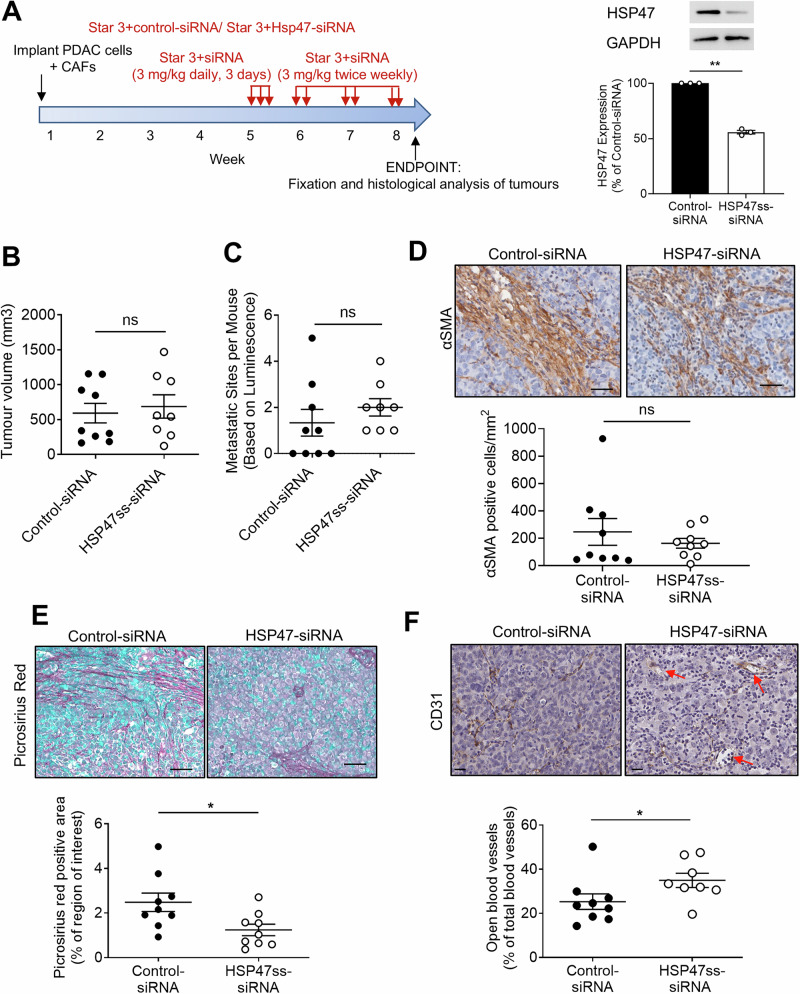
Therapeutic HSP47 knockdown in PDAC tumours reduced intratumoural fibrosis. All orthotopic tumours were co-injections of MiaPaCa-2 PDAC cells and human primary patient-derived CAFs. **A** Regimen for orthotopic tumours treated with Star 3 nanoparticles + control-siRNA or HSP47ss-siRNA. Confirmation of knockdown in vitro is also shown as quantified by qPCR (graph, *n* = 3) and representative Western blot. **B** Tumour volume at therapeutic model endpoint, as assessed by calliper measurement (*n* = 8–9). **C** Quantification of metastatic sites per mouse at model endpoint based on ex vivo luminescence (*n* = 8–9). Representative images and quantification of **D** αSMA (brown), **E** picrosirius red (collagen) and **F** CD31(brown)-stained tumour sections. Red arrows indicate open blood vessels. Bar graph shows fraction of CD31-positive vessels that were open (*n* = 8–9). Circles in panel (**A**) indicate independent CAF lines (biological replicates). Circles in all graphs in panels (**B**–**F**) represent individual mice (biological replicates). Lines in all graphs = mean ± s.e.m. Asterisks in all graphs indicate significance (*p ≤ 0.05; Student’s *t* test). Scale bars in (**D**, **E**) = 100 µm. Scale bars in (**F**) = 20 µm.

To evaluate the effect of silencing HSP47 in vivo, we used Star 3 nanoparticles to deliver HSP47ss-siRNA into the PDAC mouse tumours [25]. PDAC mouse tumours were generated by orthotopic implantation of MiaPaCa-2 PDAC cells and CAFs into the pancreas of mice and then therapeutically administered with either Star 3+control-siRNA or Star 3+HSP47ss-siRNA (Fig. 3A). These nanoparticles have previously been shown to penetrate the dense fibrotic stroma to deliver therapeutic siRNA to PDAC tumours in vivo [25, 31]. Here, treatment of PDAC mouse tumours with Star 3+HSP47ss-siRNA did not inhibit tumour growth or metastatic spread relative to controls (Fig. 3B, C). Moreover, immunohistochemical staining for α-smooth muscle actin (αSMA), a marker for myofibroblast CAFs (myCAFs; responsible for collagen deposition and remodelling), revealed comparable numbers of αSMA positive cells in control and HSP47 knockdown tumours (Fig. 3D). However, analysis of picrosirius red staining (collagen, a key component of fibrosis) showed a significant reduction in HSP47 knockdown tumours relative to control tumours (Fig. 3E). This was associated with an increased percentage of open CD31-positive blood vessels in HSP47 knockdown tumours relative to controls (Fig. 3F), implying normalization of tumour vasculature.

### Stable HSP47 knockdown specifically in the stromal compartment of PDAC tumours reduced tumour growth rate, reduced fibrosis and increased open blood vessels

**Fig. 4:**
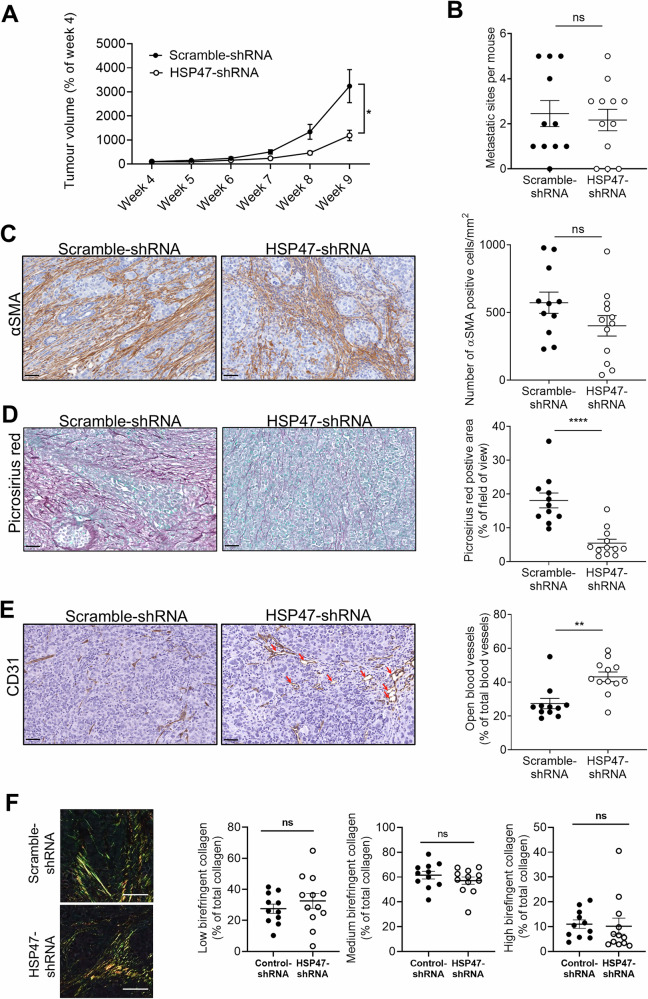
Stable HSP47 knockdown specifically in the stromal compartment of PDAC tumours reduced tumour growth rate, fibrosis, and increased open blood vessels. All orthotopic tumours were co-injections of MiaPaCa-2 PDAC cells and human primary patient-derived CAFs. **A** Line graphs show average tumour volume (as a % of week 4 tumour volume) per group, based on ultrasound measurements. Symbols show mean ± s.e.m (*n* = 11 scramble-shRNA, *n* = 12 HSP47-shRNA). **B** Tumour metastasis (sites per mouse) at model end point based on ex vivo luminescence (*n* = 11 scramble-shRNA, *n* = 12 HSP47-shRNA). Representative photos and quantification of staining for **C** αSMA (brown), **D** picrosirius red (red), and **E** CD31 (brown) in tumour tissue (*n* = 11 scramble-shRNA, *n* = 12 HSP47-shRNA). Red arrows in panel (**E**) show open blood vessels. **F** Representative photos and quantification of polarised light birefringence in stable HSP47 knockdown animal model (*n* = 11 scramble-shRNA, *n* = 12 HSP47-shRNA). Circles in all graphs signify individual mice (biological replicates). Asterisks in all graphs indicate significance (ns not significant; *p ≤ 0.05, **p ≤ 0.01; Student’s *t* test). Scale bars (**C**–**E**) = 100 μm. Scale bars (**F**) = 400 μm.

**Fig. 5:**
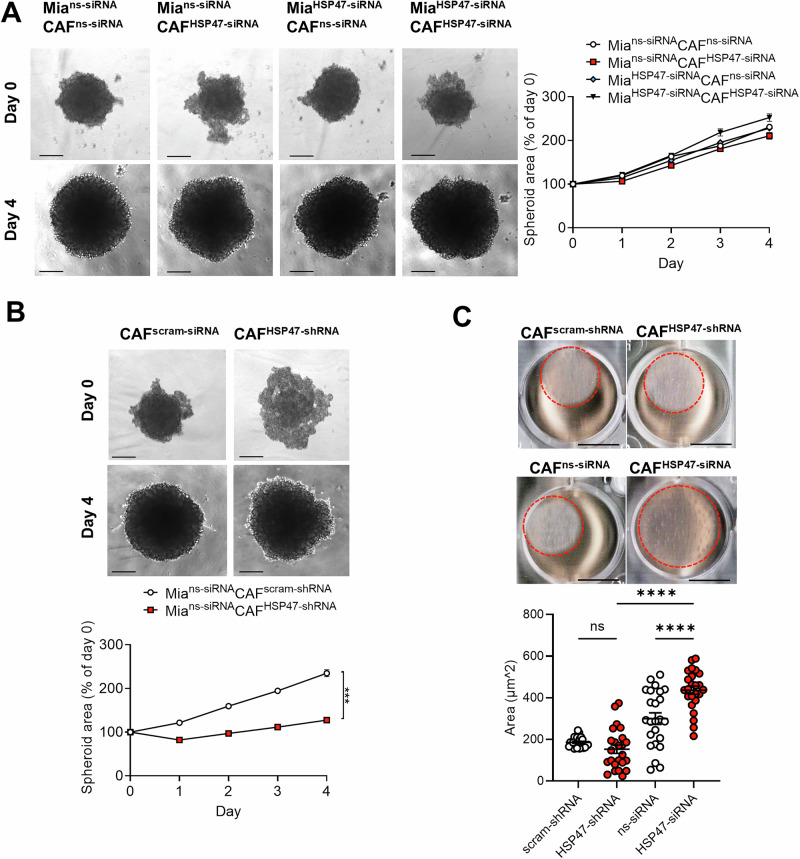
Transient versus stable HSP47 knockdown in CAFs had different impacts on PDAC 3D organoid growth and collagen remodelling in vitro. **A**, **B** MiaPaCa-2 PDAC cells were co-cultured with patient-derived CAFs as 3D organoids, embedded in a 1:1 mix of Matrigel and CAF complete culture medium. For panel (**A**), Cells were transfected with siRNA 24 h before spheroid formation. For panel (**B**), shRNA-expressing CAFs were immediately co-seeded with MiaPaCa2 cells for spheroid formation. **A** Quantification of organoid outgrowth post-transfection with control-siRNA (ns-siRNA) or HSP47-siRNA. Line graphs show spheroid cross-section area based on daily bright field microscope photos, as a percent of day 0 (*n* = 5). Labels: MiaPaCa2_ns-siRNA_, CAF_ns-siRNA_ = controls; MiaPaCa2_HSP47-siRNA_, CAF_HSP47-siRNA_ = HSP47 knockdown. Representative photos of day0 and day4 organoids are shown. Scale bars = 100 µm. Symbols and lines in graph indicate mean ± s.e.m of independent technical replicates (*n* = 5). **B** Quantification of organoid outgrowth. Line graphs show spheroid cross-section area based on daily bright field microscope photos, as a percent of day 0. Labels: CAF_scram-shRNA_ = scramble (control)-shRNA expressing CAFs co-cultured with MiaPaCa-2 cells; CAF_HSP47-shRNA_ = HSP47-shRNA expressing CAFs co-cultured with MiaPaCa-2 cells. Representative photos of day0 and day4 organoids are shown. Scale bars = 100 µm. Symbols and lines in graph indicate mean ± s.e.m of independent technical replicates (*n* = 5). **C** Representative photos and area quantification of collagen plugs contracted by siRNA-transfected CAFs over 6 days (Scale bars = 1 cm). Circles indicate independent technical replicates. Asterisks in graphs indicate significance (***p ≤ 0.001, ****p ≤ 0.0010) by (**A**, **B**) Two-way ANOVA or (**C**) One-Way ANOVA.

To delineate the contribution of CAFs to the impact of therapeutic HSP47 knockdown in PDAC tumours in vivo, we orthotopically co-implanted MiaPaCa-2 PDAC cells with immortalised CAFs stably expressing either control-shRNA or HSP47-shRNA, into the pancreas of host mice. Ultrasound tracking of tumours revealed that stable knockdown of HSP47 in CAFs substantially reduced tumour growth rate relative to controls (Fig. 4A). However, it did not affect metastatic spread (Fig. 4B). The tumours were assessed for αSMA by immunohistochemistry and results showed no significant difference between control-shRNA and HSP47-shRNA tumours (Fig. 4C). Importantly, picrosirius red staining showed that HSP47-shRNA tumours had significantly less intratumoural fibrosis than controls (69.8 ± 6.5% reduction; Fig. 4D), consistent with our therapeutic knockdown model findings. Likewise, immunohistochemical analysis of CD31 showed a significantly higher number of open blood vessels in the stable HSP47 knockdown tumours, relative to controls (57.6 ± 10.4% increase; Fig. 4E). The results were not associated with any change in the fraction of low, medium and high-density collagen fibrils (Fig. 4F) implying these effects were purely associated with overall reduced collagen deposition. The contrast in anti-tumour efficacy between therapeutic knockdown and stable knockdown models led us to assess whether this was due to inherent changes in CAFs with extended stable inhibition of HSP47, or simply due to knockdown of HSP47 from tumour establishment. We used 3D organoid co-cultures of PDAC cells and CAFs with/without transient HSP47 knockdown or stable HSP47 knockdown, using the same cell lines employed in Figs. 3 and 4. We observed that transient or stable knockdown of HSP47 in CAFs resulted in significantly looser spheroids forming at day 0 (Fig. 5A, B). However, transient knockdown in CAFs alone or in both CAFs and PDAC cells did not affect organoid growth (Fig. 5A). In contrast, stable knockdown of HSP47 in CAFs significantly reduced organoid growth (Fig. 5B) indicating that the differences we observed in vivo may be due to extended HSP47 knockdown selecting CAFs that have reduced ability to support tumour growth. This difference was also observed in a collagen contraction assay, which measures the capacity of CAFs to remodel collagen (Fig. 5C). In this setting, CAFs stably expressing HSP47-shRNA were still able to remodel collagen to the same extent as control CAFs, while transient knockdown of HSP47 significantly reduced collagen contraction relative to controls (Fig. 5C).

### HSP47 is abundant in the stroma of most human PDAC patients but its expression is independently prognostic only in the tumour compartment

**Fig. 6:**
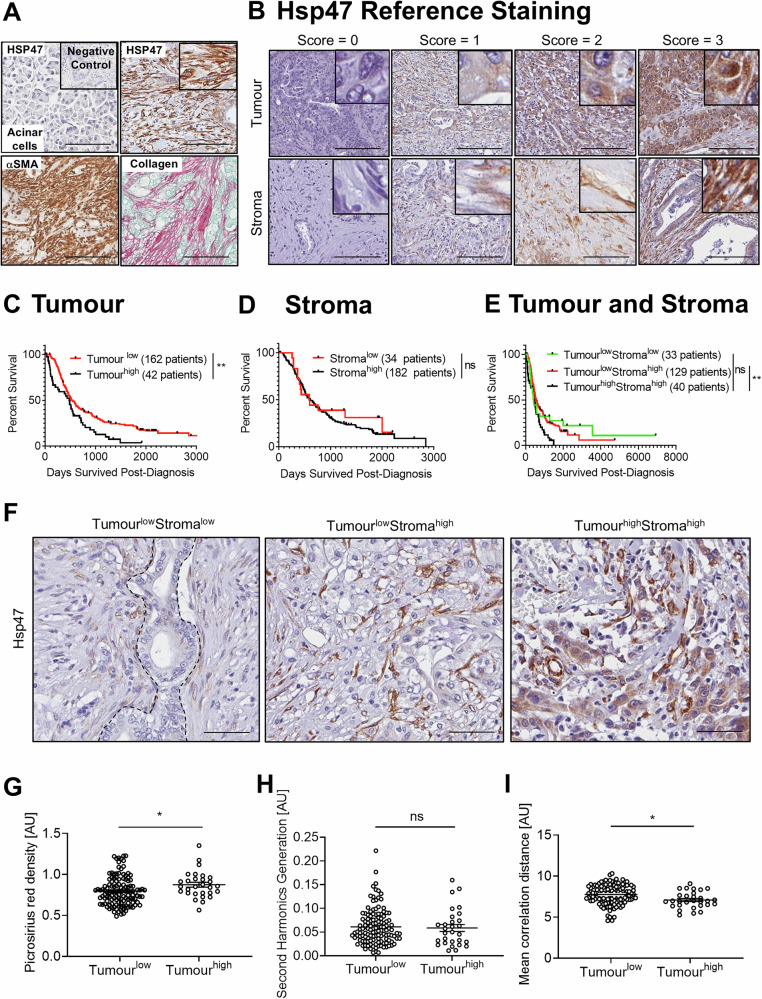
High HSP47 expression in the PDAC tumour compartment of PDAC patients is prognostic of poorer overall survival. **A** Representative images of co-localisation of HSP47, αSMA, and collagen in human PDAC specimens (APGI Training cohort). Negative control and HSP47 in acinar cells are also shown for reference. **B**–**E** Human PDAC tissue microarrays (APGI International Cancer Genome Consortium and Training cohort) were stained for HSP47. **B** Reference images for scoring tumour and stroma (insets show magnified view). Scores of 0–1 = Hsp47 low (Tumour^low^ or Stroma^low^), scores of 2–3 = Hsp47 high (Tumour^high^ or Stroma^high^). Kaplan–Meier survival curves showing the correlation between Hsp47 expression in **C** tumour, **D** stroma, and **E** combined tumour+stroma with overall patient survival (days post-diagnosis). Total patient numbers per group are indicated in keys. Asterisks indicate significance based on multivariate analysis and log-rank test (**p ≤ 0.01). **F** Representative images of combined tumour and stroma score groups. Dashed lines delineate tumour and stroma. Scale bars in all images = 100 μm. Analyses of ICGC cohort based on **G** picrosirius red (collagen) density, **H** collagen organization and directionality through second harmonics generation and **I** mean correlation distance as determined by GLCM analysis. Circles indicate individual patients. Lines indicate mean ± s.e.m. Asterisks indicate significance (ns not significant; *p ≤ 0.05).

The prognostic significance of base levels of HSP47 was assessed in both tumour and stromal compartments in human PDAC tissue microarrays obtained from the APGI International Cancer Genome Consortium (ICGC) and training cohorts (Table 2). We showed that HSP47 co-localised with αSMA and collagen but was absent in normal acinar cells (>90% of pancreas in health, Fig. 6A). We then stained human PDAC tissue microarrays by immunohistochemistry and analysed HSP47 expression in the tumour and stromal compartments. We scored the HSP47 expression using independent scoring scales for tumour and stroma (Fig. 6B) and correlated with overall patient survival (Fig. 6C–F). HSP47 was highly expressed in the tumour compartment of 21% of patients (Fig. 6C) and in the stromal compartment of 84% of patients (Fig. 6D). Multivariate analysis showed that while high HSP47 in the stroma did not correlate with survival (likely due to the low number of Stroma^low^ patients), high HSP47 in the tumour compartment was independently prognostic of poorer overall survival (*p* = 0.014, Hazard ratio = 1.571; Fig. 6C, Table 2). Combined high HSP47 expression in tumour and stromal compartments was significantly predictive of poorer overall patient survival compared to the Tumour^low^Stroma^high^ group (Fig. 6E, F). Given the prognostic significance of HSP47 in the tumour compartment, and HSP47’s role as a collagen chaperone, we assessed whether tumour cell expression of HSP47 correlated with tumour fibrosis. We aligned data generated by Vennin et al. [33] and Murphy et al. [7] on total picrosirius red content, second harmonic generation and collagen organisation (GLCM) in the APGI ICGC cohort. Results showed that Tumour^high^ patients had significantly higher picrosirius red content relative to the Tumour^low^ group (Fig. 6G) and while no significant difference was observed in fibrillar collagen detected by second harmonics generation (Fig. 6H), we did observe significantly poorer collagen organisation in the Tumour^high^ group relative to Tumour^low^ (Fig. 6I).

**Table 2:** HSP47 multivariate survival analysis parameters.

Univariate parameter	HR	95%	CI	Univariate (log-rank) *p*-value
Tumour score	1.571	1.095	2.255	0.014
Stroma score	1.271	0.829	1.950	0.272
Gender	0.845	0.631	1.132	0.260
Age at diagnosis (years)	0.718	0.439	1.174	0.187
Smoker	1.008	0.747	1.360	0.959
Alcohol consumption	0.962	0.709	1.306	0.804
Margin status	1.554	1.135	2.127	0.006
Lymph nodes involved	1.520	1.081	2.136	0.016
Perineural invasion	1.201	0.792	1.821	0.389
Overall stage AJCC	1.612	1.133	2.293	0.008
Macroscopic tumour location	0.861	0.516	1.439	0.569
**Multivariate parameter**	**HR**	**95%**	**CI**	**Multivariate (Cox regression)** ***p***-**value**
Tumour score	1.528	1.057	2.209	0.024
Margin status	1.453	1.048	2.015	0.025
Overall stage AJCC	1.528	1.052	2.218	0.026

Univariate parameters and their corresponding univariate Log-Rank results are shown in the top rows. Multivariate (Cox regression) analysis results are shown in the bottom three rows for the most significant parameters.

*HR* Hazard ratio, *CI* Confidence interval.

### HSP47 knockdown in a 3D human PDAC explant model reduced tumour cell frequency

**Fig. 7:**
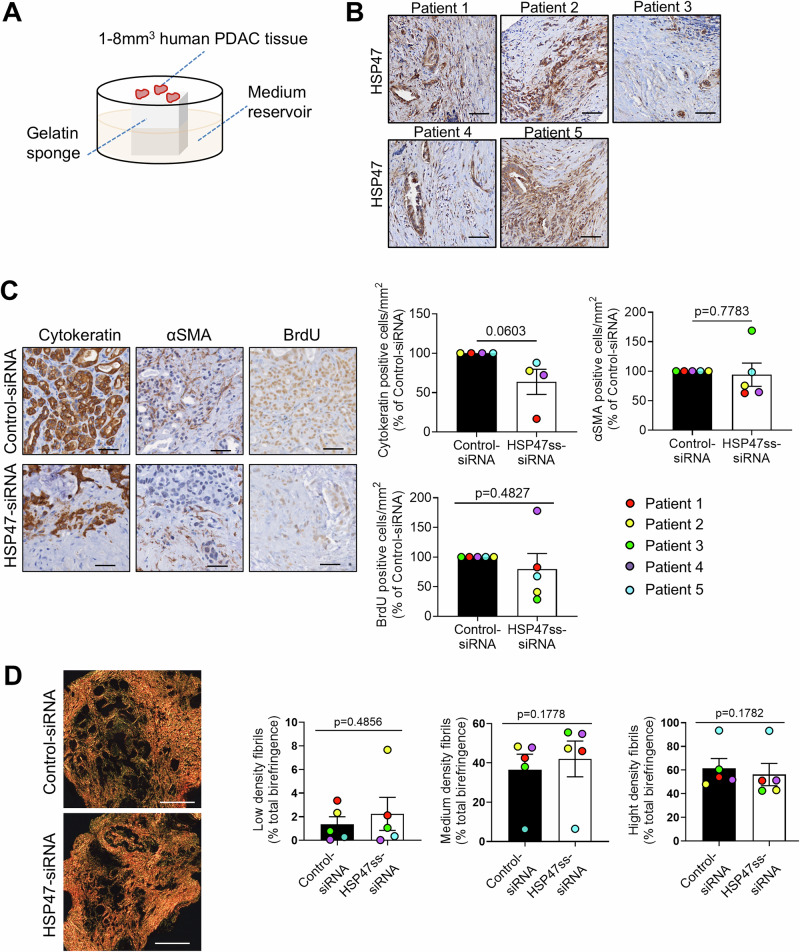
HSP47 knockdown in HSP47 Tumour^high^Stroma^high^ PDAC patient explants reduces tumour cell frequency and proliferation. **A** Schematic diagram of explant culture set up. Explants from five PDAC patient tumours (2–4 explants per sponge) were treated with Star 3+control-siRNA or Star 3+HSP47ss-siRNA then fixed and analysed by immunohistochemistry. **B** Representative photos of five human PDAC patient (patient 1–5) explants stained for HSP47 by immunohistochemistry at day 0. **C** Representative immunohistochemistry photos, and quantification (*n* = 5 patients) for cytokeratin (tumour cells), αSMA (CAFs), and BrdU (proliferation) (photos also included in per patient data, Supplementary Figs. 3–7). **D** Representative photos and quantification (*n* = 5 patients) of polarised light birefringence of treated explants (photos also included in Supplementary Fig. 8). Bars and lines indicate mean ± s.e.m. Symbols and colour indicates independent patients. (ns not significant). Scale bars (**B**, **C**) = 40μm. Scale bars (**E**) = 200μm.

We tested the functional significance of HSP47 in 3D patient-derived tumour explants [31, 34] (Fig. 7A). HSP47 knockdown in five patients with high tumour cell expression of HSP47 (Fig. 7B; Supplementary Fig. 2) reduced proliferation (BrdU) across explants in 4/5 patients, reduced αSMA-positive CAFs in 3/5 patients, and notably reduced tumour cells (cytokeratin) in 4/4 patients (Fig. 7C; Supplementary Figs. 3–7 for individual patient results; 1 patient excluded due to lack of tumour cells in explants; note that patient 1 in Supplementary Fig. 3 shares the same control explants as patient 3 in prior publication [35]). We observed only minimal changes in collagen structure (Fig. 7D, Supplementary Fig. 8), possibly due to the short time-frame of the model and presence of established collagen network. It should be noted that across all five patients combined, only changes in cytokeratin positive PDAC cells approached significance (*p* = 0.0603) and that the above findings are exploratory given the limited number of patient samples. Our findings indicate that at least in a subset of patients, we observe comparable effects to those observed in our in vivo models and form the foundation for future studies in expanded cohorts.

## Discussion

Stromal remodelling approaches offer opportunities to improve drug access and thus chemosensitivity in PDAC tumours. The collagen chaperone HSP47 has been demonstrated to play a role in tumour fibrosis as well as tumour cell survival and metastatic spread in a variety of cancers [36]. In this study, we demonstrated that HSP47 inhibition is a potential dual cell targeting approach in PDAC, with the capability to directly inhibit CAF and PDAC cell survival, and to interfere with intratumoural collagen deposition. These effects appeared to be dependent on the setting in which HSP47 knockdown was applied and were likely influenced by microenvironmental queues and the heterogeneity of both PDAC cells and CAFs. Importantly, we also showed that HSP47 expression in the tumour compartment is independently prognostic of patient survival and that it may also play an important survival role in subsets of tumour cells.

We silenced HSP47 in human patient-derived PDAC CAFs and demonstrated that not only does it inhibit collagen production (consistent with its well-established role in correct assembly of collagen fibrils [14]), but it also directly affects the proliferation and survival of CAFs. This may have been a consequence of misfolded collagen causing ER stress, which has been demonstrated in HSP47 mouse embryos [37] and in hepatic stellate cells [38]. These collagen aggregates in the endoplasmic reticulum require autophagy to clear them without inducing apoptosis [37–39]. It is possible that at least in vitro, this could have been the cause for the apoptosis induced in CAFs, but additional anti-proliferative mechanisms may also be involved. For example, Han et al. [21] were able to induce quiescence in PDAC CAFs in vitro in response to HSP47 knockdown. However, this may have also been a response to the co-treatment with all-trans retinoic acid (ATRA) [21].

We also observed that HSP47 knockdown induces anti-proliferative effects in PDAC cells in vitro and importantly, we demonstrated that in human PDAC explants, HSP47 knockdown reduced the frequency of cytokeratin-positive PDAC cells. Some explants also showed a reduction in the frequency of αSMA positive CAFs, although there was greater patient heterogeneity and less pronounced than anti-tumour cell and anti-proliferative effects. Our results are indicative of a direct pro-survival role for HSP47 in PDAC cells. We acknowledge that patient samples were limited and that these trends are at this stage exploratory. Our findings form the foundation for future larger scale validation studies to further delineate the role of HSP47 in each cellular compartment. This is consistent with prior findings that HSP47 plays a functional survival role in a variety of other cancer cell types including breast, glioma and colon [22, 40–42]. For example, HSP47 expression in breast cancer cells is required for invasion [42] and the production of surface collagen that allows clustering with platelets and facilitates metastatic spread [40]. HSP47 knockdown in glioma cells was found to reduce their growth, invasion and migration in vitro and tumour growth in vivo [41]. Yoneda et al. [22] demonstrated that many cancer cell lines, including a number of PDAC cell lines, express HSP47, and that knockdown of HSP47 compromised their survival by inducing ER stress [22]. The same group subsequently showed that HSP47 is a chemoresistance factor in PDAC cells that can inhibit the unfolded protein response by interacting with calreticulin and IRE1α [23].

In an in vivo setting, stable HSP47-shRNA expression in an immortalised PDAC CAF cell line in vivo was able to reduce orthotopic PDAC tumour growth and intratumoural fibrosis, and increased the frequency of open intratumoural blood vessels, but did not affect intratumoural CAF frequency or metastatic spread. We observed similar results with therapeutic delivery of HSP47ss-siRNA to orthotopic PDAC tumours in mice, despite a lack of effect on tumour growth in this setting. In this latter setting, it is also possible that indirect effects of HSP47 inhibition in tumour cells interfering with CAF cross-talk could have also contributed to this effect. Our therapeutic model findings are consistent with observations by Han et al. [21], which utilised an inorganic nanoparticle to deliver HSP47ss-siRNA to orthotopic and sub-cutaneous PDAC tumours in mice. In contrast to this prior study, our nanoparticle system is organic and does not require co-treatment with ATRA for uptake [25]. Taken together, our in vivo findings together with our 3D organoid and collagen contraction assay results suggest the difference in anti-tumour growth effects between stable knockdown (shRNA) and therapeutic knockdown (siRNA) of HSP47 could have been a consequence of changes induced by long-term selection of a subset of CAFs that could tolerate extended HSP47 knockdown in the shRNA setting. Our results imply that extended HSP47 therapeutic inhibition may inhibit tumour growth in addition to interfering with collagen deposition which was observed in both transient and stable knockdown settings. A limitation of our study is that direct causality of CAF-specific HSP47 inhibition was not definitively established in a therapeutic model, and that comparisons between stable and transient knockdown based approaches need to be validated with an orthogonal approach. Taken together with our in vitro and ex vivo findings, our work suggests that both cancer cell and CAF compartments can contribute to the observed in vivo phenotype induced by HSP47 knockdown. To address this limitation, future studies should assess individual compartment contributions in vivo, with inducible lineage-specific knockout models.

What was consistent across both in vivo models is that we observed no change in the frequency of αSMA-positive CAFs, suggesting that in an in vivo setting, the primary benefit of HSP47 inhibition is to reduce intratumoural fibrosis and normalise tumour vasculature, without necessarily reducing CAF survival or proliferation. Han et al. [21] used gold nanoparticles to deliver HSP47-siRNA to PDAC tumours in an orthotopic mouse model of the disease. The group found that while HSP47 knockdown alone did not affect tumour growth, it substantially reduced intratumoural fibrosis and coupled with vitamin-A treatment, was able to sensitise PDAC tumours to gemcitabine [21]. The potential for nanomedicine mediated reprogramming of desmoplasia to enhance drug penetration and efficacy has also been demonstrated across a variety of highly desmoplastic solid tumours [43]. Future work will definitively assess whether HSP47 inhibition using our nanoparticle system similarly enhances drug permissiveness of pancreatic tumours, using combination therapy in vivo models.

It should be noted that there have also been conflicting studies on the role of tumour fibrosis in promoting versus inhibiting PDAC invasion and metastasis. Chen et al. [10] demonstrated that deletion of collagen I from αSMA positive CAFs accelerated PDAC metastasis and enhanced immune suppression. Bhattacharjee et al. [44] similarly showed that collagen I deletion in hepatic stellate cells that are converted to CAFs by metastasising cancer cells, increased PDAC and colon cancer metastases to the liver, implying fibrosis also plays a role in hindering tumour growth at metastatic sites. These findings also come after multiple studies that demonstrated CAF (and CAF-generated fibrosis) ablation from PDAC tumour initiation can accelerate tumour growth and metastases [10–13]. However, the key difference between our therapeutic approach and these studies is that we reduced collagen deposition rather than depleted tumour fibrosis. Moreover, by targeting a collagen chaperone we do not target a single collagen sub-type but rather interfere with the maturation and folding of all collagen from these cells. We also need to take into account the variety of functionally distinct subsets of CAFs that have been identified (broadly myCAFs, iCAFs, apCAFs [45]). Transcriptomic analysis in PDAC CAF subsets by Tao et al. [46] detected upregulated SERPINH1 (HSP47) in nCAFs (broad precursor cells to CAF subsets) relative to normal fibroblasts (log2FC = 1.706), and higher expression in classical myCAFs over all other CAF subsets (log2FC = 1.159). It would not be unexpected that HSP47 might be playing a more prominent role in ECM-producing myCAFs, but that does not rule out its functional relevance in other CAF subsets. It is also possible that HSP47 may induce reprogramming between CAF subsets, particularly with sustained HSP47 inhibition, but this requires further investigation to confirm.

Finally, we assessed the prognostic significance of HSP47 in tumour and stromal elements in human PDAC tissue microarrays from the APGI ICGC cohort. Consistent with prior studies [17–19], we observed abundant HSP47 in the stromal compartment of 84% of PDAC patients. As a consequence of the small number of stromal HSP47 low tumours, we did not have the power to demonstrate if this was prognostic of PDAC patient survival. However, we found that high HSP47 in the tumour compartment (20% of all patients) predicts poorer overall patient survival. This was particularly evident when tumour and stromal scores were combined, where patients that had high HSP47 expression in both the tumour and stromal compartments had significantly poorer overall survival relative to those which only had high expression in the stroma. This is the first study to demonstrate that HSP47 expression specifically in the tumour compartment of any cancer predicts patient survival. Importantly, patients that had high HSP47 expression in the tumour compartment also had higher collagen content (fibrosis) with poorer organisation. What remains unclear is whether the relationship between high HSP47 expression in the tumour compartment and tumour fibrosis is causative. While this will be the subject of future studies, our findings may indicate a crucial role for HSP47 in PDAC cell tumourigenicity in a subset of PDAC patients, and that PDAC tumour cells may also participate in the development and/or remodelling of tumour fibrosis. Our findings come as Chen et al. [24] demonstrated in a transgenic mouse model of the disease that production of homotrimeric collagen I specifically by PDAC cells plays a pro-tumour role through interactions with α3β1 integrin on PDAC cells, modulation of the tumour microbiome and inhibition of effector T cell infiltration.

Our study reinforces the role of HSP47 in deposition of mature collagen from tumour initiation, but suggests that HSP47 needs to be combined with a stromal remodelling agent to degrade existing collagen matrix, and a chemotherapy to enhance anti-tumour effects. Our findings also highlight the dual therapeutic targeting potential of this approach against PDAC cells. Importantly, we describe a potential new therapeutic approach for pancreatic cancer using a biodegradable nanoparticle that targets HSP47, to alleviate physical barriers to drug delivery and to directly inhibit PDAC cells.

## Supplementary information

Supplementary Figures

Supplementary Full Western Blots

## Data Availability

All data is included within this manuscript and upon request.
