# Supplementary material for: HSP47 is a potential dual cell target and prognostic factor in pancreatic cancer
Source: Oncogene. 2026 Jun 21;45(31):3165–80. doi: 10.1038/s41388-026-03865-y (PMC13407167; doi:10.1038/s41388-026-03865-y)
Supplement: Supplementary file 1 — Supplementary Figures [file 41388_2026_3865_MOESM1_ESM.pdf]

Supplementary Figures: HSP47 is a potential dual cell target and prognostic factor in pancreatic cancer

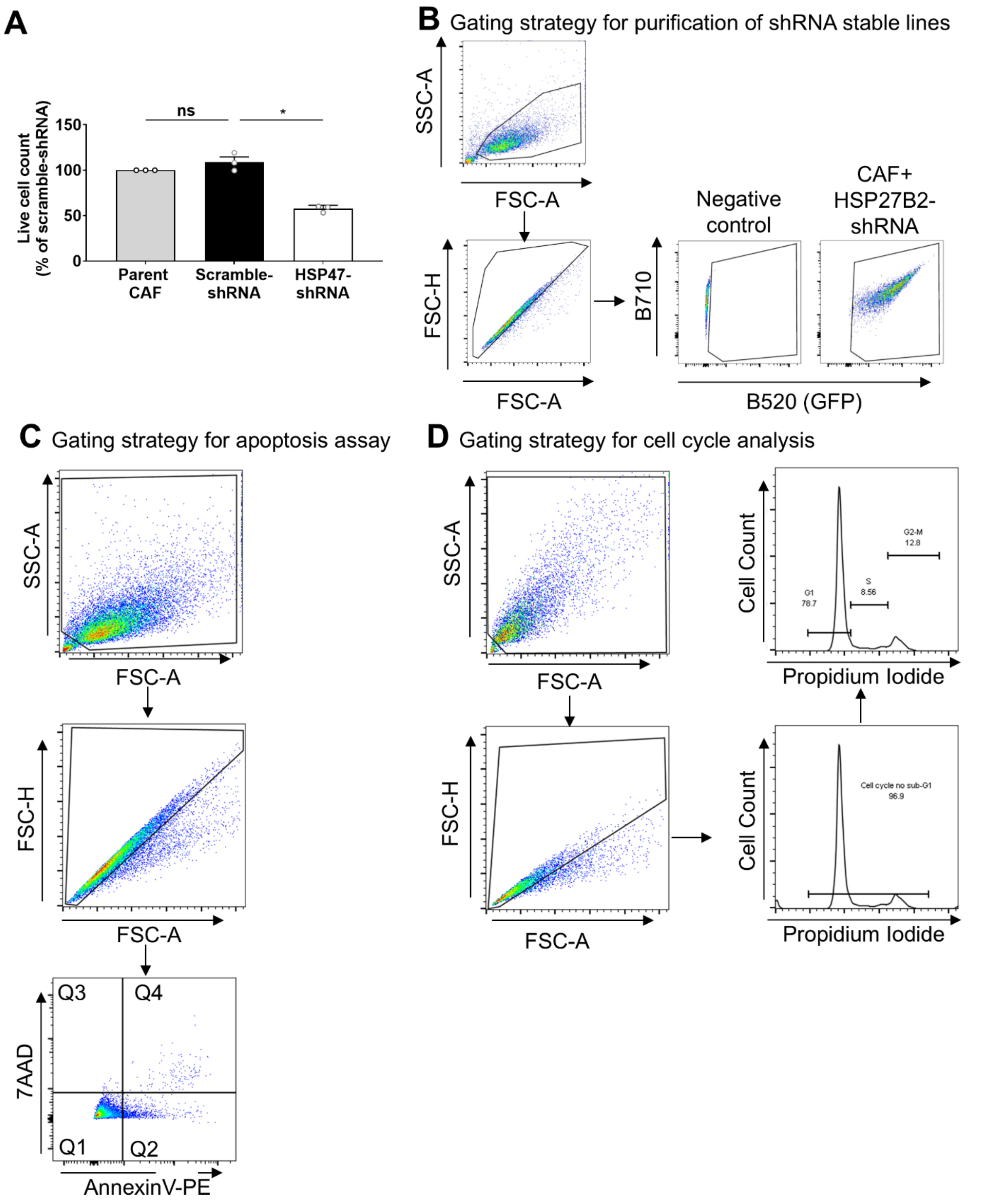

Supplementary Figure 1: Cell sorting and flow cytometry gating strategies, proliferation and polarised light birefringence of hTERT immortalised CAFs stably expressing shRNA. A) Live cell counts (trypan blue exclusion assay) of hTERT-immortalised CAFs stably expressing control(scramble)-shRNA or HSP47-shRNA, relative to matched, non-immortalised CAFs (parent CAF), 72h post-seeding. Circles represent independent experiments with the same cell line. Bars and lines = mean+s.e.m. Asterisks indicate

significance (ns=not significant; *p≤0.05; One-way ANOVA; n=3). **B-D)** Panels show representative flow cytometry plots demonstrating the gating strategy for **(B)** stable-shRNA line cell sorting (final plots show an example of a negative vs sorted population), **(C)** apoptosis analysis and **(D)** cell cycle analysis. FSC-A/H = forward scatter area/height, SSC = side scatter.

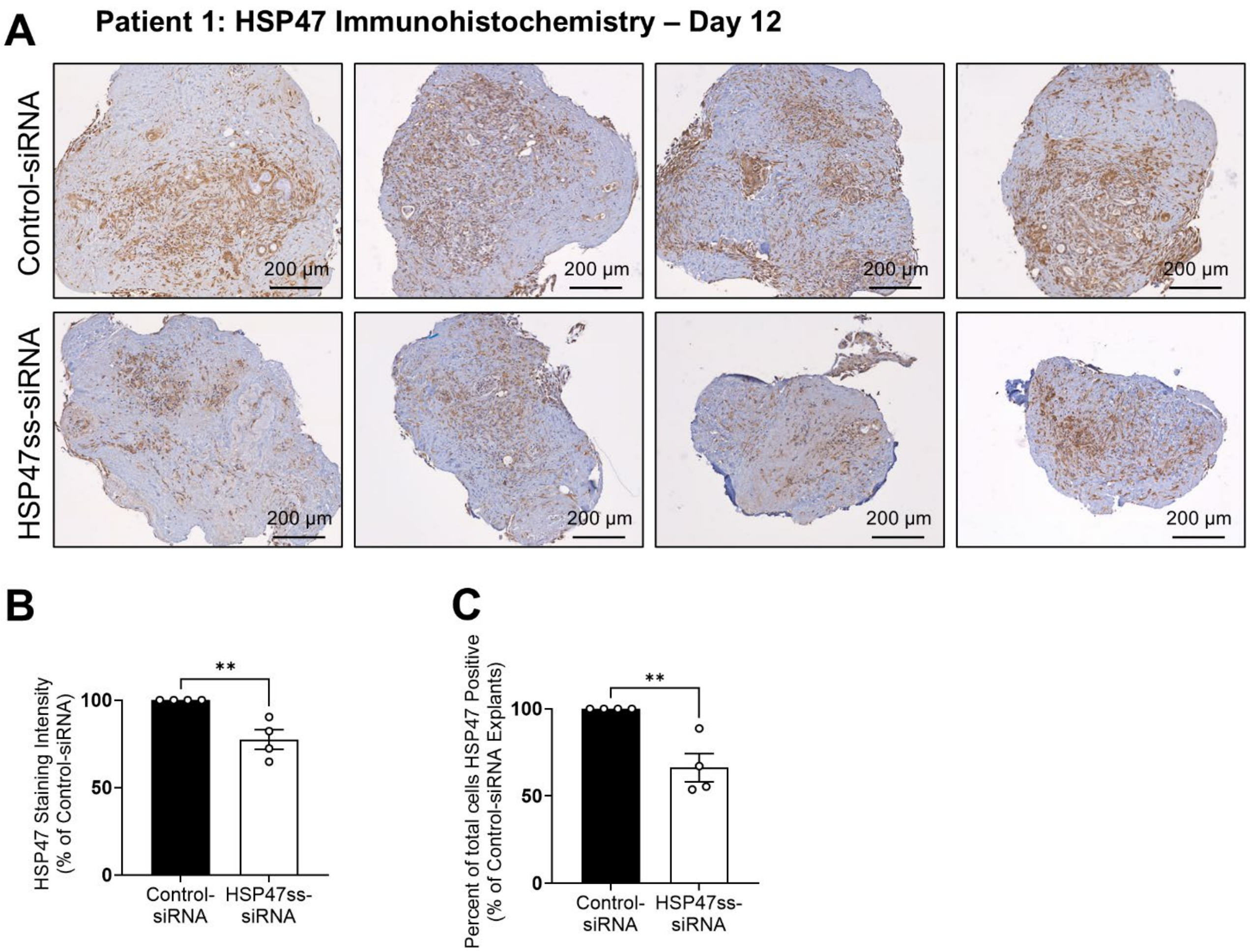

Supplementary Figure 2: Confirmation of HSP47 knockdown in PDAC patient explants treated with Star 3+HSP47ss-siRNA. A) Representative photos of immunohistochemistry staining for HSP47 in 4x explants from a PDAC patient with high HSP47 expression in tumour and stroma, at day 12 of culture and after 4 rounds of treatment with Star 3+control-siRNA or Star 3+HSP47ss-siRNA. Graphs show quantification of **(B)** HSP47 staining intensity and **(C)** the frequency of HSP47 positive cells. Bars and lines indicate mean ± s.e.m. Symbols indicate individual explants from the same patient (n=4). Asterisks and p-values indicate significance based on student t-test (**p≤0.01).

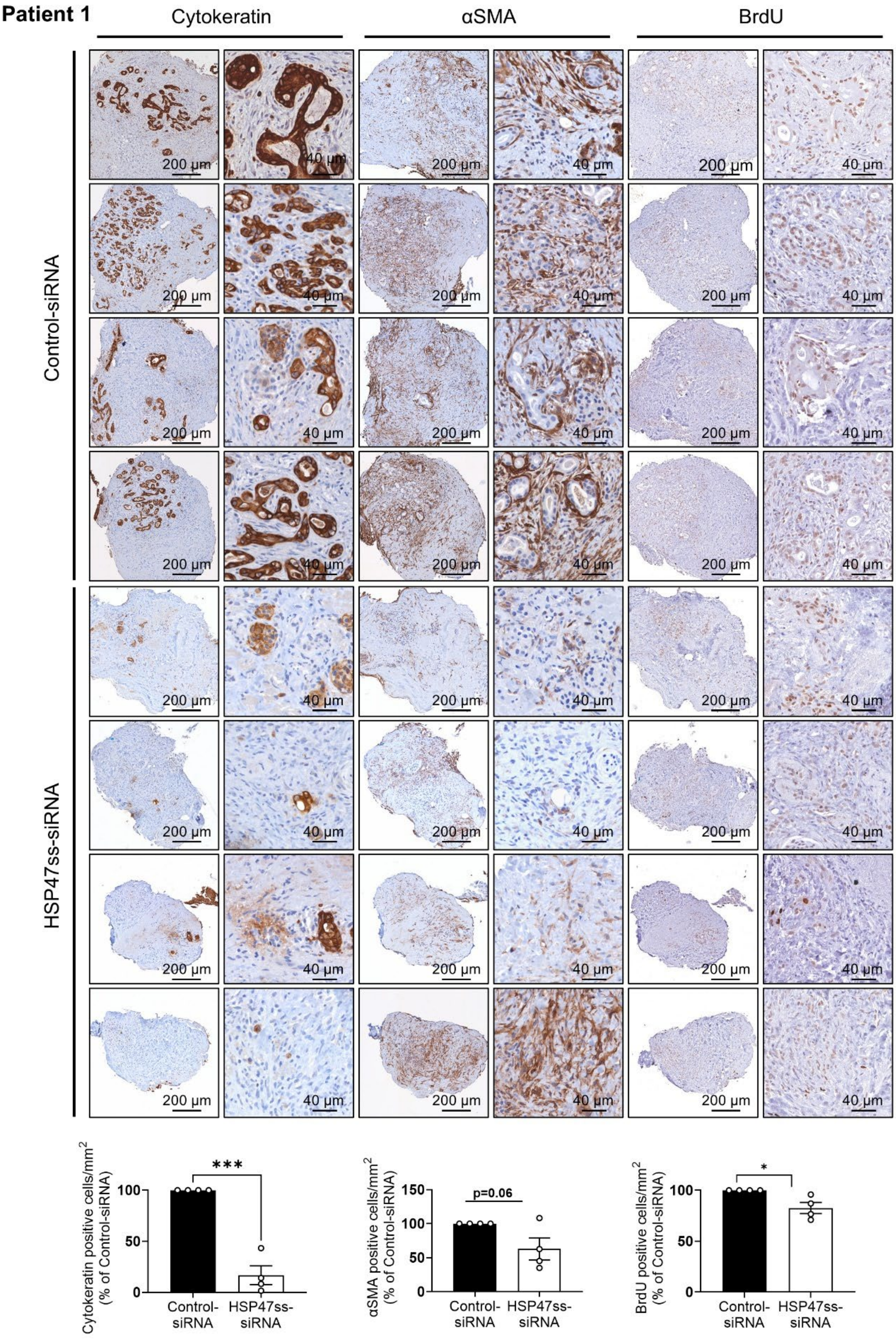

Supplementary Figure 3: Immunohistochemistry photos and quantification for tumour and stromal markers in PDAC patient 1 explants. Photos and quantification of

immunohistochemistry staining for cytokeratin (tumour cells), αSMA (CAFs), and BrdU (proliferation) in explants from patient 1 at day 12 of culture and after four rounds of treatment with Star 3+control-siRNA or Star 3+HSP47ss-siRNA. Symbols indicate individual explants from the same patient. Asterisks and p-values indicate significance based on student t-test (* $p \leq 0.05$,*** $p \leq 0.001$). Please note that the control treatments for patient 1 were shared with experiments also presented for patient 3 in BioRxiv (doi: https://doi.org/10.1101/2022.09.29.510034) and cell death and disease [33] (data modified to be expressed as % of matched regions) under creative commons BY licence:

https://s100.copyright.com/AppDispatchServlet?title=%CE%B2III-tubulin%20can%20act%20as%20a%20brake%20on%20extrinsic%20apoptosis%20in%20pancreatic%20cancer&author=George%20Sharbeen%20et%20al&contentID=10.1038%2Fs41419-026-08657-6&copyright=The%20Author%28s%29&publication=2041-4889&publicationDate=2026-04-24&publisherName=SpringerNature&orderBeanReset=true&oa=CC%20BY.

Patient 2

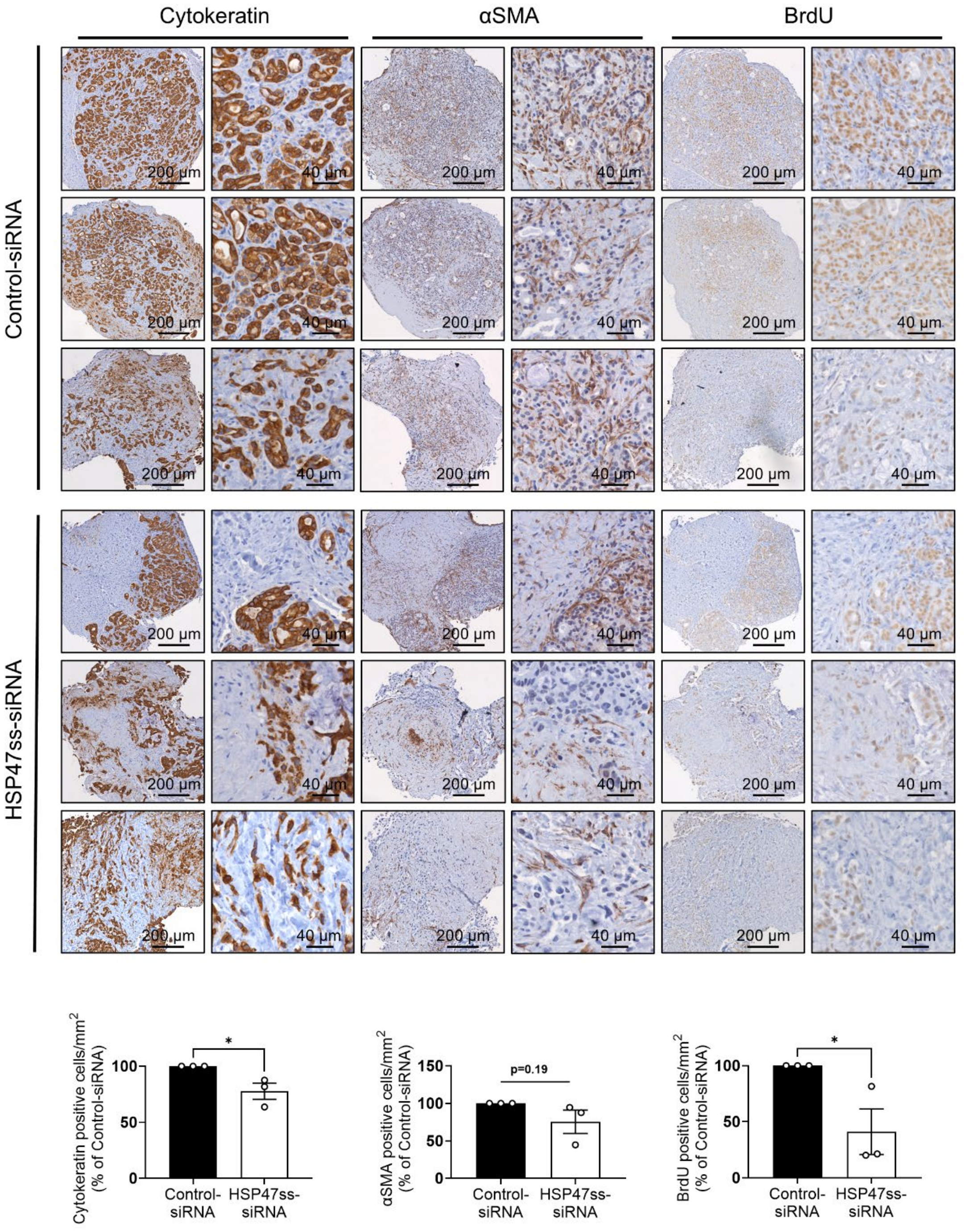

Supplementary Figure 4: Immunohistochemistry photos and quantification for tumour and stromal markers in PDAC patient 2 explants. Photos and quantification of immunohistochemistry staining for cytokeratin (tumour cells), αSMA (CAFs), and BrdU (proliferation) in explants from patient 1 at day 12 of culture and after four rounds of treatment with Star 3+control-siRNA or Star 3+HSP47ss-siRNA. Symbols indicate individual explants

from the same patient. Asterisks and p-values indicate significance based on student t-test (*p≤0.05).

Patient 3

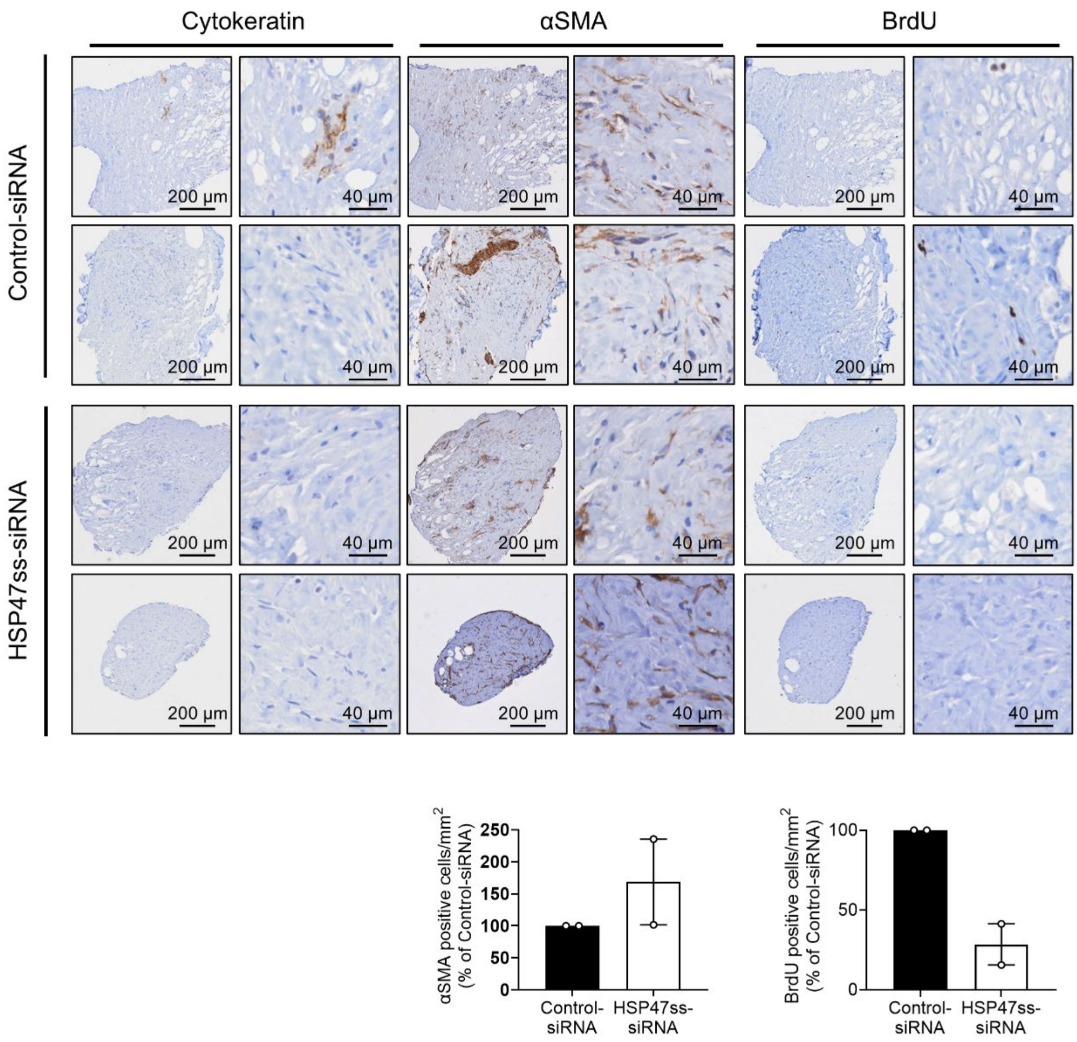

Supplementary Figure 5: Immunohistochemistry photos and quantification for tumour and stromal markers in PDAC patient 3 explants. Photos and quantification of immunohistochemistry staining for cytokeratin (tumour cells), αSMA (CAFs), and BrdU (proliferation) in explants from patient 1 at day 12 of culture and after four rounds of treatment with Star 3+control-siRNA or Star 3+HSP47ss-siRNA. Symbols indicate individual explants from the same patient. Note that there were not enough tumour cells to quantify in this patient.

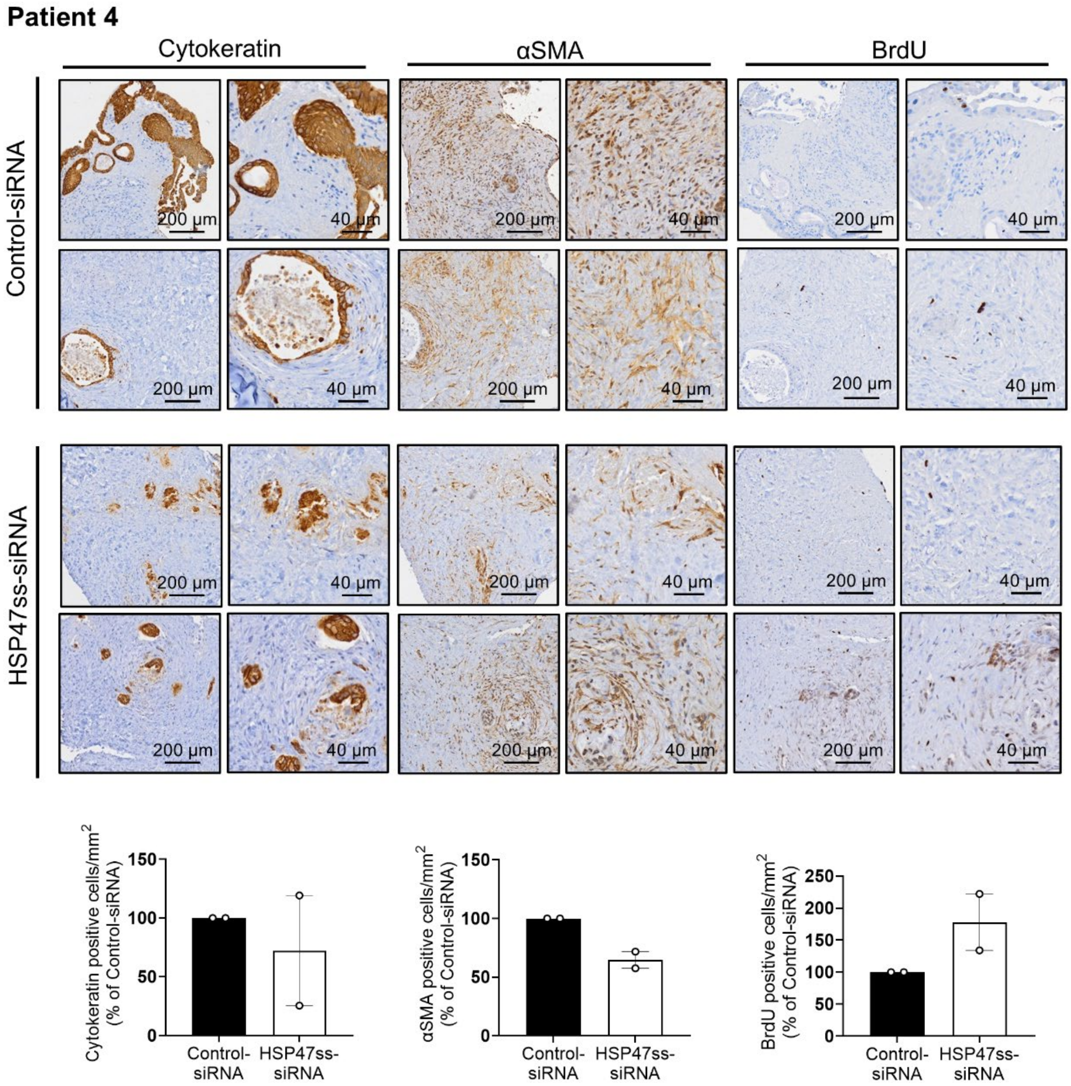

Supplementary Figure 6: Immunohistochemistry photos and quantification for tumour and stromal markers in PDAC patient 4 explants. Photos and quantification of immunohistochemistry staining for cytokeratin (tumour cells), αSMA (CAFs), and BrdU (proliferation) in explants from patient 1 at day 12 of culture and after four rounds of treatment with Star 3+control-siRNA or Star 3+HSP47ss-siRNA. Symbols indicate individual explants from the same patient.

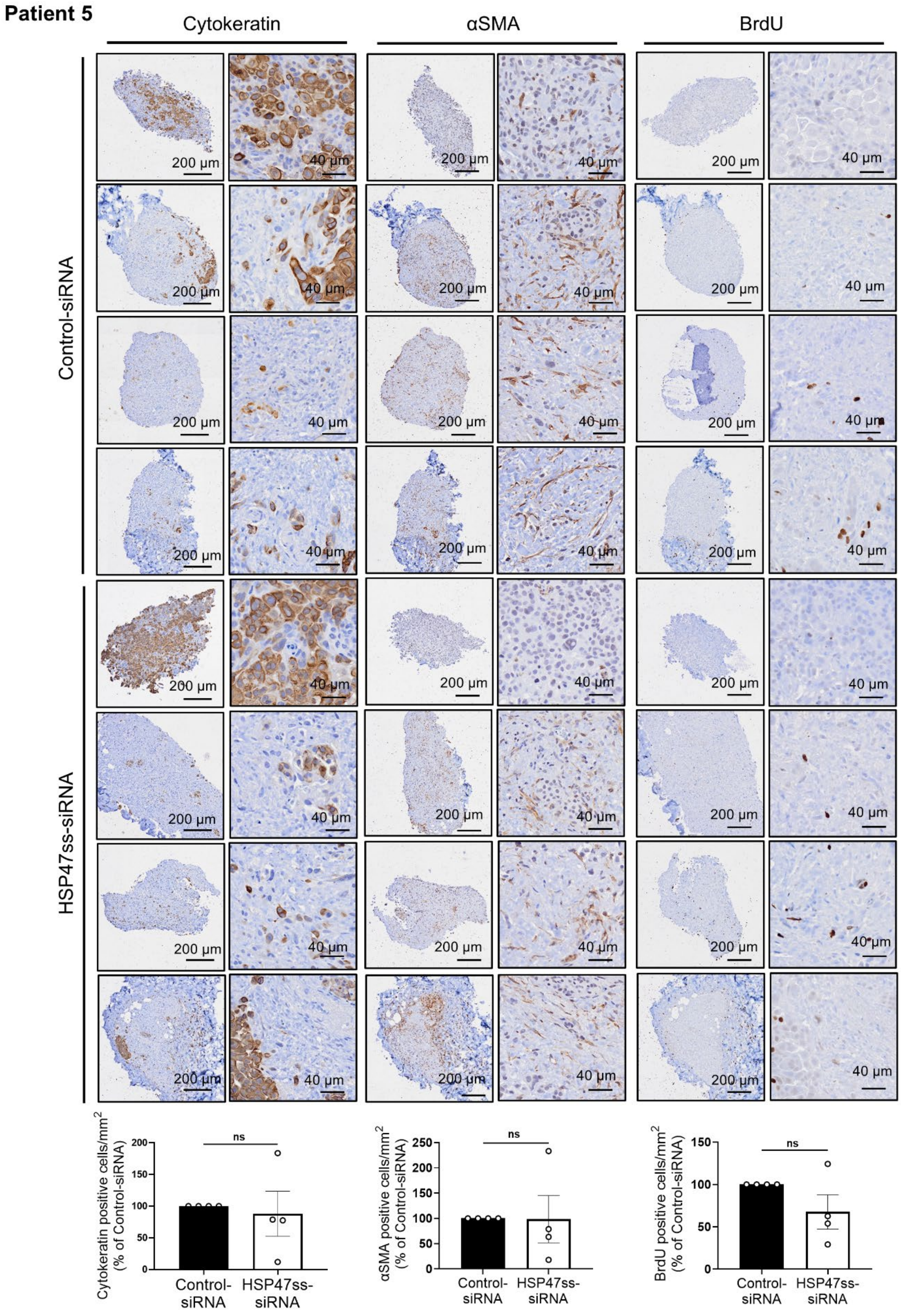

Supplementary Figure 7: Immunohistochemistry photos and quantification for tumour and stromal markers in PDAC patient 5 explants. Photos and quantification of

immunohistochemistry staining for cytokeratin (tumour cells), αSMA (CAFs), and BrdU (proliferation) in explants from patient 1 at day 12 of culture and after four rounds of treatment with Star 3+control-siRNA or Star 3+HSP47ss-siRNA. Symbols indicate individual explants from the same patient. Asterisks and p-values indicate significance based on student t-test (ns=not significant).

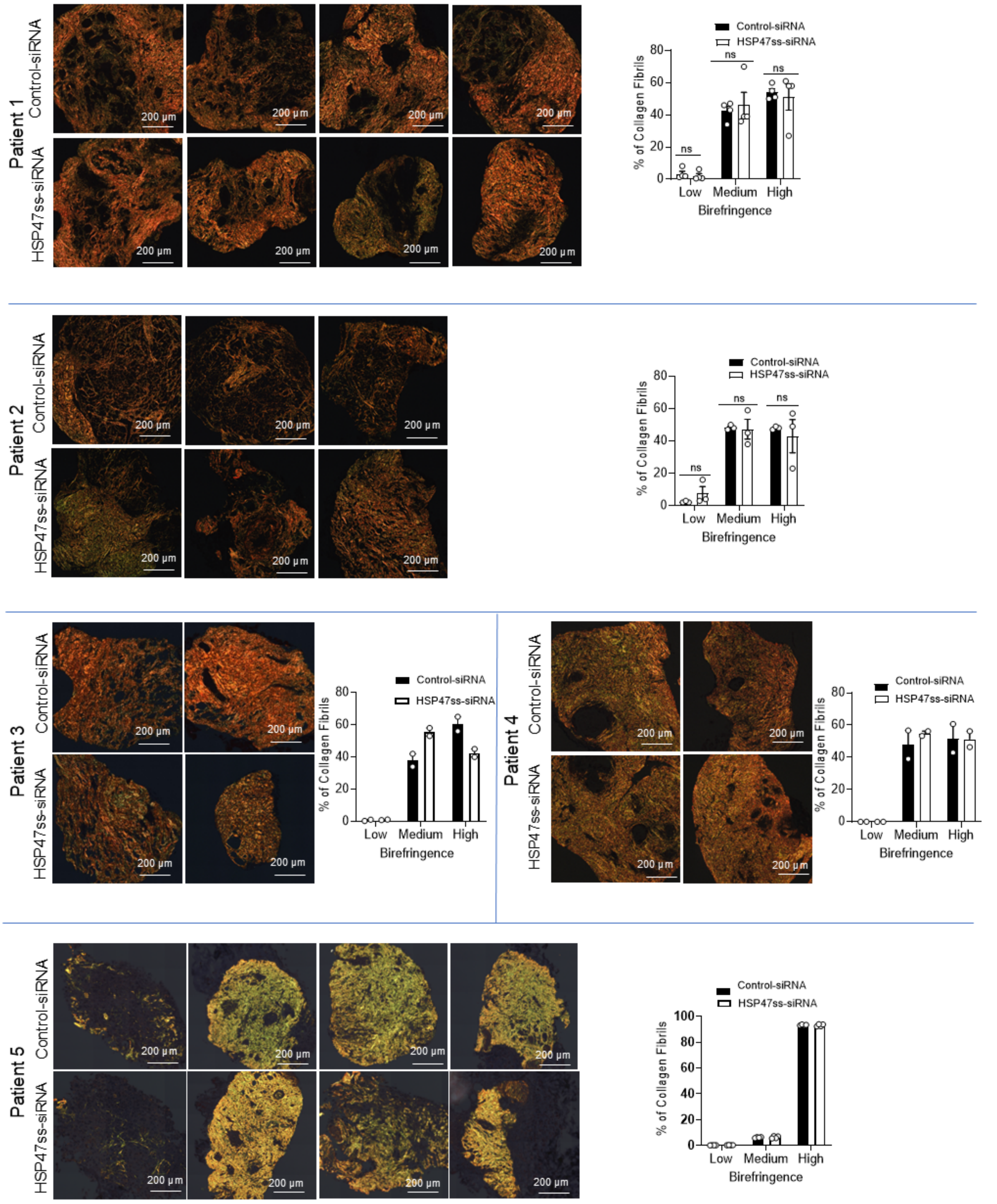

Supplementary Figure 8: Polarised light birefringence analysis of collagen in explants from five HSP47 PDAC patients treated with Star 3+HSP47-siRNA. (A) Polarised light photos and (B) quantification of low (green, low density collagen fibrils), medium (yellow, intermediate density collagen fibrils) and high birefringence (highly dense collagen fibrils), per patient. Measurements performed on tissue sections from day 12 of culture and after four

rounds of treatment with Star 3+control-siRNA or Star 3+HSP47-siRNA. Symbols indicate individual explants form the same patient. (ns = not significant).

Australian Pancreatic Cancer Genome Initiative Consortium

Garvan Institute of Medical Research Amber L. Johns[1], Anthony J Gill[1,5], Lorraine A. Chantrill[1,22], Paul Timpson[1,] Angela Chou[1,5], Marina Pajic[1], Tanya Dwarte[1], David Herrmann[1], Claire Vennin[1], Thomas R Cox[1], Brooke Pereira[1], Shona Ritchie[1], Daniel A Reed[1], Cecilia R Chambers[1], Xanthe Metcalf[1], Max Nobis[1], Gloria Jeong[1], Ruth J. Lyons[1]. **QIMR Berghofer Medical Research Institute** Nicola Waddell[2], John V. Pearson[2], Ann-Marie Patch[2], Katia Nones[2], Felicity Newell[2], Pamela Mukhopadhyay[2], Venkateswar Addala[2], Stephen Kazakoff[2], Oliver Holmes[2], Conrad Leonard[2], Scott Wood[2]. **University of Melbourne, Centre for Cancer Research** Sean M. Grimmond[3], Oliver Hofmann[3]. **Royal North Shore Hospital** Jaswinder S. Samra[5], Nick Pavlakis[5,] Jennifer Arena[5], Hilda A. High[5]. **Bankstown Hospital** Ray Asghari[6], Neil D. Merrett[6], Amitabha Das[6]. **Liverpool Hospital** Peter H. Cosman[7], Kasim Ismail[7]. **St Vincent's Hospital** Alina Stoita[8], David Williams[8], Allan Spigellman[8]**. Westmead Hospital** Duncan McLeod[9], Judy Kirk[9]. **Royal Prince Alfred Hospital, Chris O'Brien Lifehouse** James G. Kench[10], Peter Grimison[10], Charbel Sandroussi[10], Annabel Goodwin[7,10]. **Prince of Wales Hospital** R. Scott Mead[1,11], Katherine Tucker[11], Lesley Andrews[11]. **Fiona Stanley Hospital** Michael Texler[12], Cindy Forrest[12], Mo Ballal[12,13], David Fletcher[12]**. St John of God Healthcare** Maria Beilin[13], Kynan Feeney[13] Krishna Epari[13] Sanjay Mukhedkar[13]. **Epworth HealthCare** Nikolajs Zeps[23]. **Royal Adelaide Hospital** Nan Q Nguyen[14], Andrew R. Ruszkiewicz[14], Chris Worthley[14]. **Flinders Medical Centre** John Chen[15], Mark E. Brooke-Smith[15], Virginia Papangelis[15]. **Envoi Pathology** Andrew D. Clouston[16]. **Princess Alexandra Hospital** Andrew P. Barbour[17], Thomas J. O'Rourke[17], Jonathan W. Fawcett[17], Kellee Slater[17], Michael Hatzifotis[17], Peter Hodgkinson[17]. **Austin Hospital** Mehrdad Nikfarjam[18]. **Johns Hopkins Medical Institutes** James R. Eshleman[19], Ralph H. Hruban[19], Christopher L. Wolfgang[19]. **ARC-Net Centre for Applied Research on Cancer** Aldo Scarpa[20], Rita T. Lawlor[20], Vincenzo Corbo[20], Claudio Bassi[20]. **University of Glasgow** Andrew V Biankin [21], Nigel B. Jamieson[21] David K. Chang[1, 21,] Stephan B. Dreyer[21].

[1]The Kinghorn Cancer Centre, Garvan Institute of Medical Research, 370 Victoria Street, Darlinghurst, Sydney, New South Wales 2010, Australia.

[2]QIMR Berghofer Medical Research Institute, 300 Herston Rd, Herston, Queensland 4006, Australia.

[3]University of Melbourne, Centre for Cancer Research, Victorian Comprehensive Cancer Centre, 305 Grattan Street, Melbourne, Victoria 3000, Australia.

[5]Royal North Shore Hospital, Westbourne Street, St Leonards, New South Wales 2065, Australia.

[6]Bankstown Hospital, Eldridge Road, Bankstown, New South Wales 2200, Australia.

[7]Liverpool Hospital, Elizabeth Street, Liverpool, New South Wales 2170, Australia.

[8] St Vincent's Hospital, 390 Victoria Street, Darlinghurst, New South Wales, 2010 Australia.

[9]Westmead Hospital, Hawkesbury and Darcy Roads, Westmead, New South Wales 2145, Australia.

[10]Royal Prince Alfred Hospital, Missenden Road, Camperdown, New South Wales 2050, Australia.

[11]Prince of Wales Hospital, Barker Street, Randwick, New South Wales 2031, Australia.

[12]Fremantle Hospital, Alma Street, Fremantle, Western Australia 6959, Australia.

[13] St John of God Healthcare, 12 Salvado Road, Subiaco, Western Australia 6008, Australia.

[14] Royal Adelaide Hospital, North Terrace, Adelaide, South Australia 5000, Australia.

[15] Flinders Medical Centre, Flinders Drive, Bedford Park, South Australia 5042, Australia.

[16] Envoi Pathology, 1/49 Butterfield Street, Herston, Queensland 4006, Australia.

[17] Princess Alexandra Hospital, 199 Ipswich Rd, Woolloongabba QLD 4102

[18] Austin Hospital, 145 Studley Road, Heidelberg, Victoria 3084, Australia.

[19] Johns Hopkins Medical Institute, 600 North Wolfe Street, Baltimore, Maryland 21287, USA.

[20] ARC-NET Center for Applied Research on Cancer, University of Verona, Via dell'Artigliere, 19 37129 Verona, Province of Verona, Italy.

[21] Wolfson Wohl Cancer Research Centre, Institute of Cancer Sciences, University of Glasgow, Garscube Estate, Switchback Road, Bearsden, Glasgow, Scotland G61 1BD, United Kingdom.

[22] Wollongong Hospital, Illawarra and Shoalhaven Local Health District, Loftus Street, Wollongong NSW 2500.

[23] Epworth HealthCare, 89 Bridge Rd, Richmond VIC 3121, Australia
