# Supplementary material for: HSP47 is a potential dual cell target and prognostic factor in pancreatic cancer
Source: Oncogene. 2026 Jun 21;45(31):3165–80. doi: 10.1038/s41388-026-03865-y (PMC13407167; doi:10.1038/s41388-026-03865-y)
Supplement: Supplementary file 2 — Supplementary Full Western Blots [file 41388_2026_3865_MOESM2_ESM.pdf]

Figure 1

A

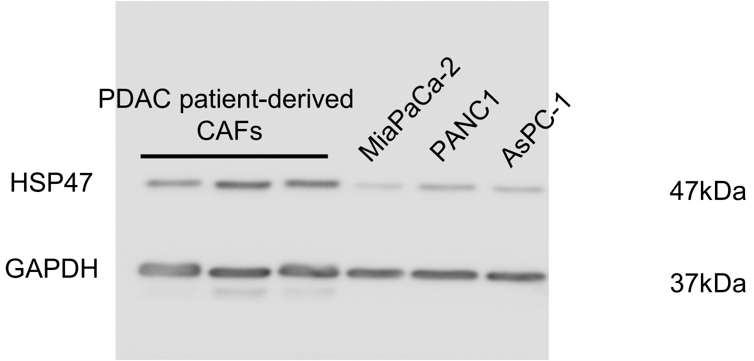

Note GAPDH and HSP47 were stained separately as strips from the same blot, but imaged together in the single photo above

Figure 1

C

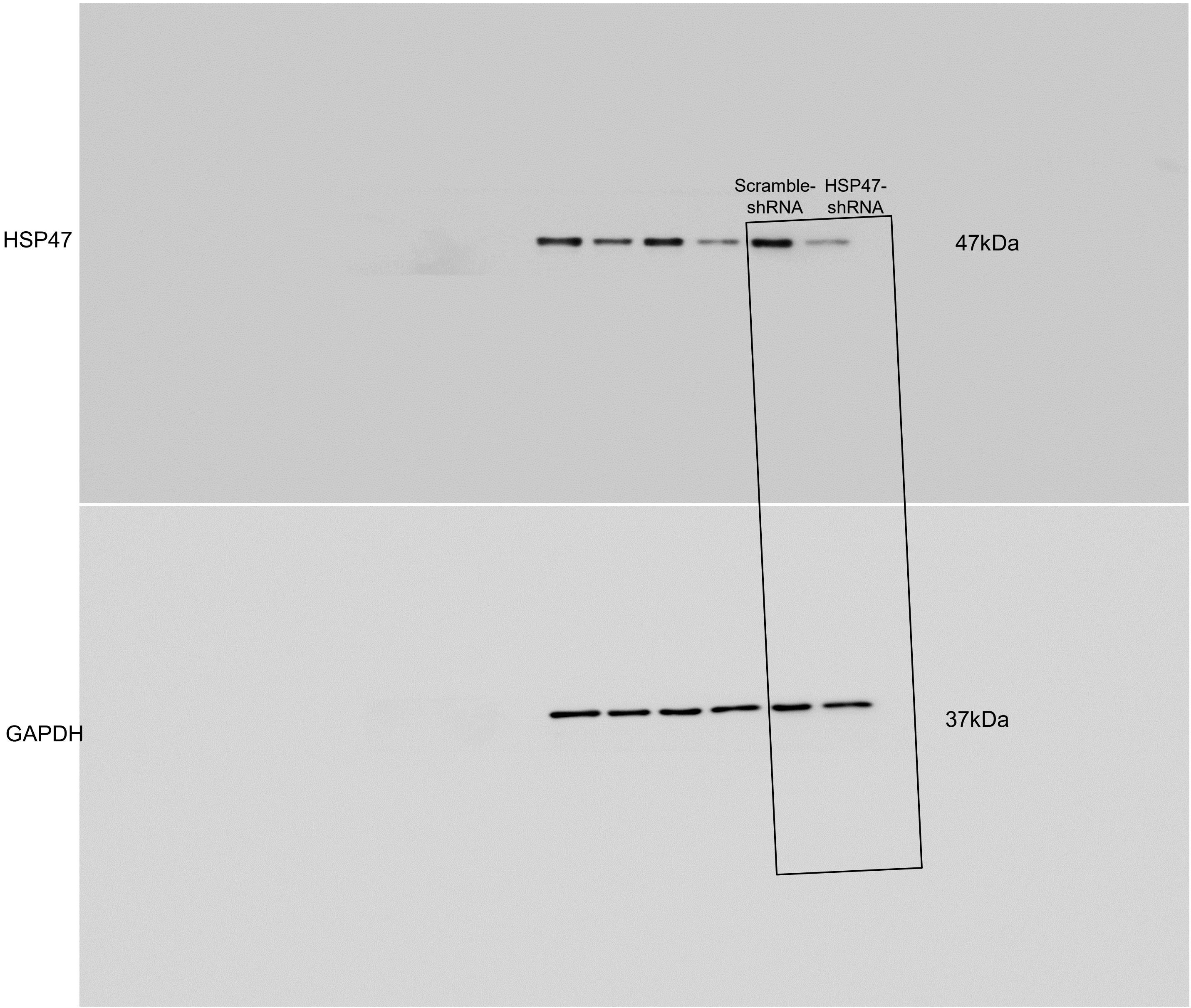

Figure 1

E

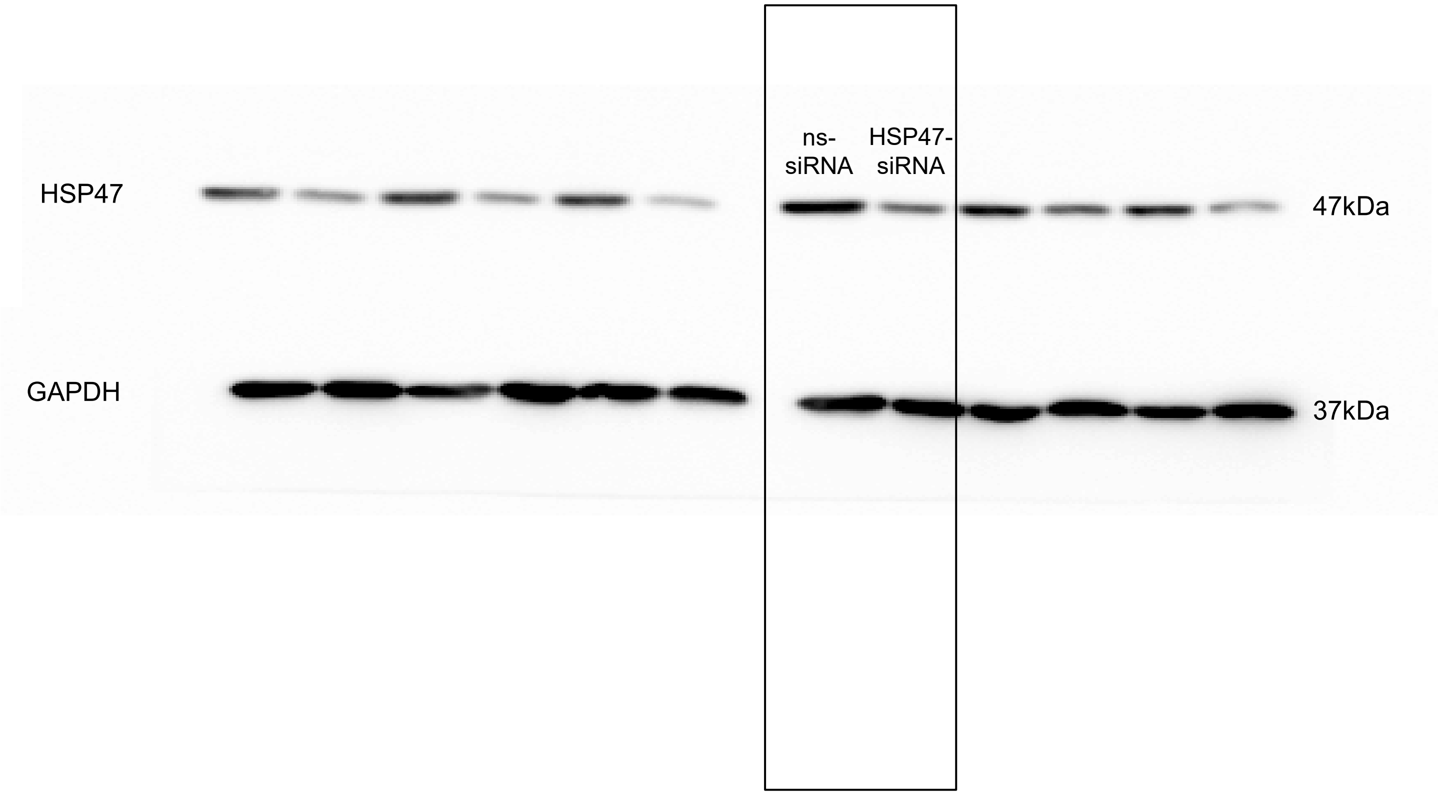

Figure 2

D MiaPaCa2

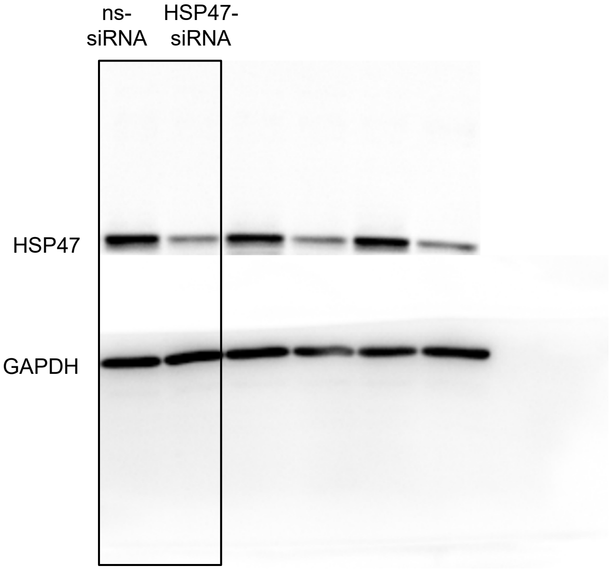

F AsPC1

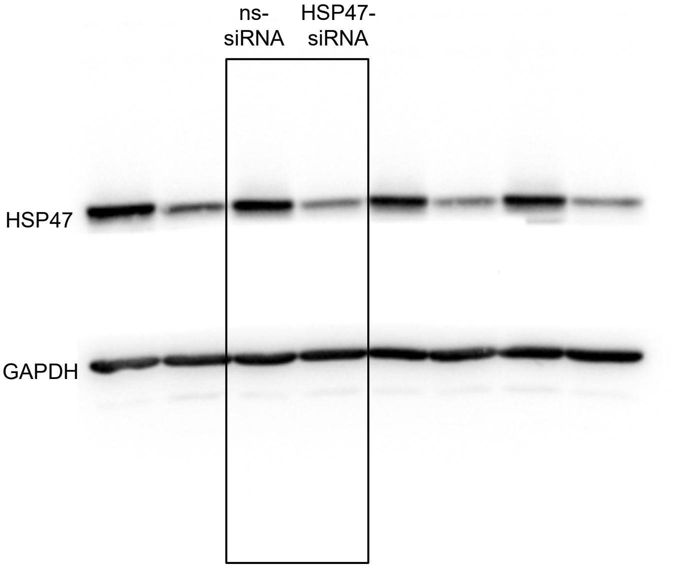

Figure 3

A

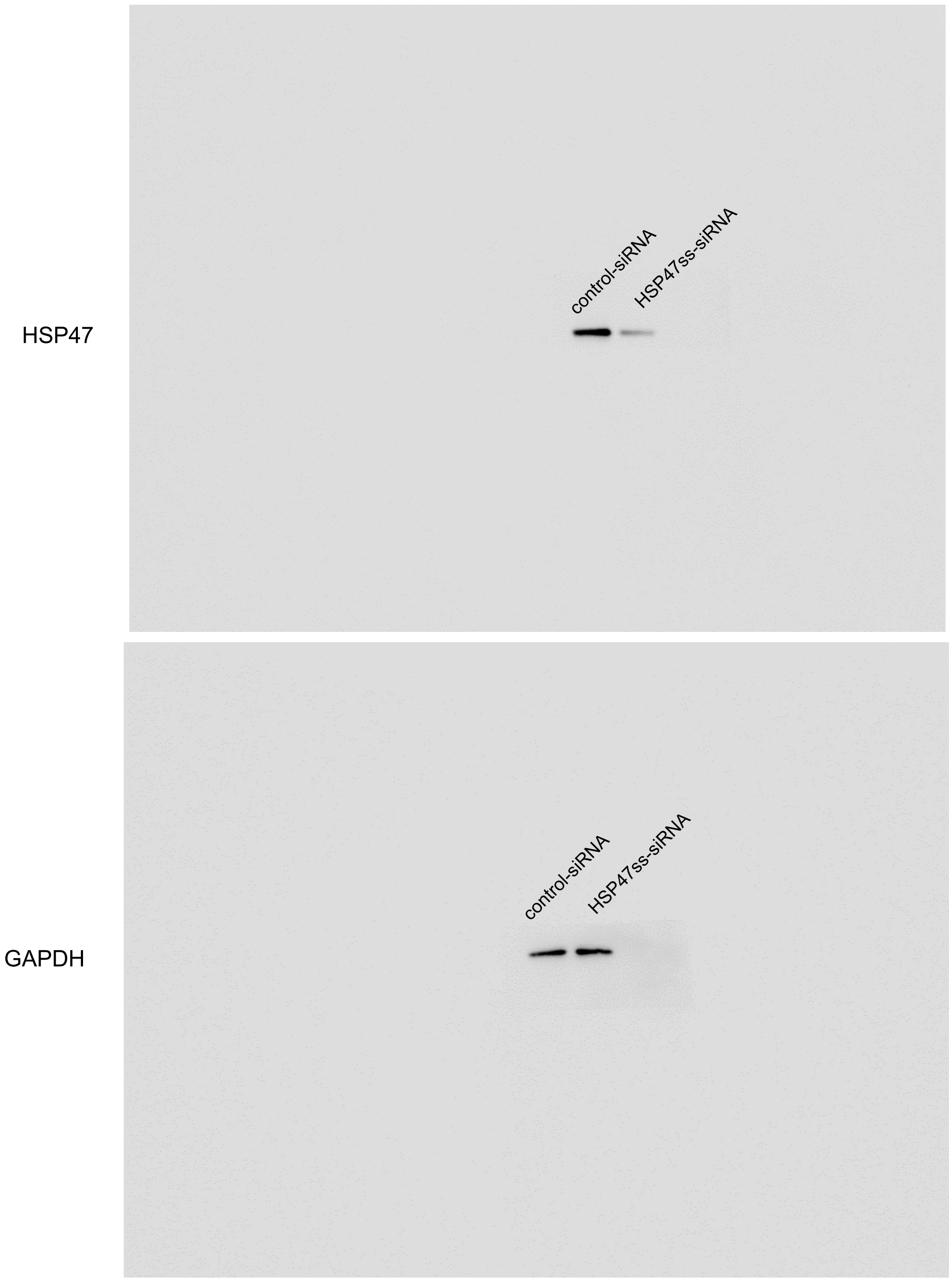

HSP47
control-siRNA
HSP47ss-siRNA
GAPDH
control-siRNA
HSP47ss-siRNA
